# Heterogeneous combinatorial expression of *Hoxd* genes in single cells during limb development

**DOI:** 10.1186/s12915-018-0570-z

**Published:** 2018-09-18

**Authors:** P. J. Fabre, M. Leleu, B. Mascrez, Q. Lo Giudice, J. Cobb, D. Duboule

**Affiliations:** 1 School of Life Sciences, Ecole Polytechnique Fédérale, Lausanne, 1015 Lausanne, Switzerland; 2 Department of Genetics and Evolution, University of Geneva, 1211 Geneva 4, Switzerland; 3 Department of Biological Sciences, University of Calgary, Calgary, Canada; 4 Department of Basic Neurosciences, University of Geneva, 1211 Geneva, Switzerland

**Keywords:** *Hox* genes, Digits, Limb, Development, Enhancers, Single-cell, Transcriptome, Differentiation, Gene expression

## Abstract

**Background:**

Global analyses of gene expression during development reveal specific transcription patterns associated with the emergence of various cell types, tissues, and organs. These heterogeneous patterns are instrumental to ensure the proper formation of the different parts of our body, as shown by the phenotypic effects generated by functional genetic approaches. However, variations at the cellular level can be observed within each structure or organ. In the developing mammalian limbs, expression of *Hox* genes from the *HoxD* cluster is differentially controlled in space and time, in cells that will pattern the digits and the forearms. While the *Hoxd* genes broadly share a common regulatory landscape and large-scale analyses have suggested a homogenous *Hox* gene transcriptional program, it has not previously been clear whether *Hoxd* genes are expressed together at the same levels in the same cells.

**Results:**

We report a high degree of heterogeneity in the expression of the *Hoxd11* and *Hoxd13* genes. We analyzed single-limb bud cell transcriptomes and show that *Hox* genes are expressed in specific combinations that appear to match particular cell types. In cells giving rise to digits, we find that the expression of the five relevant *Hoxd* genes (*Hoxd9* to *Hoxd13*) is unbalanced, despite their control by known global enhancers. We also report that specific combinatorial expression follows a pseudo-time sequence, which is established based on the transcriptional diversity of limb progenitors.

**Conclusions:**

Our observations reveal the existence of distinct combinations of *Hoxd* genes at the single-cell level during limb development. In addition, we document that the increasing combinatorial expression of *Hoxd* genes in this developing structure is associated with specific transcriptional signatures and that these signatures illustrate a temporal progression in the differentiation of these cells.

**Electronic supplementary material:**

The online version of this article (10.1186/s12915-018-0570-z) contains supplementary material, which is available to authorized users.

## Background

**Fig. 1:**
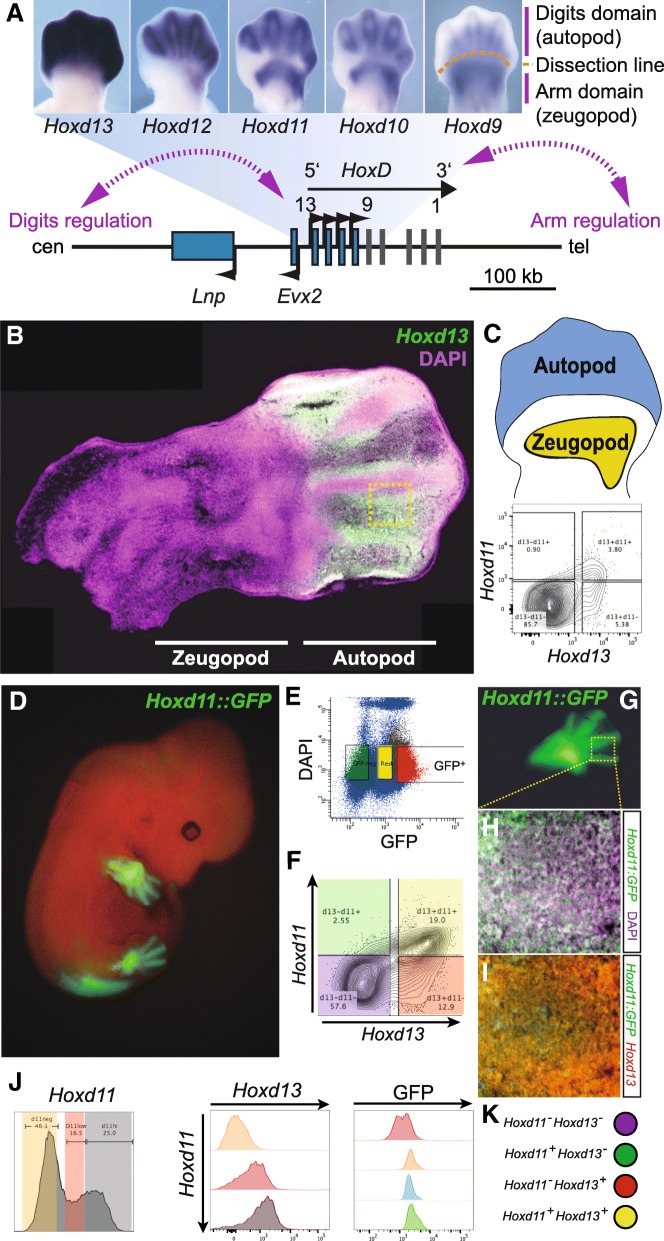
Heterogeneity of *Hoxd* mRNAs in single limb bud cells. **a** Scheme representing the organization of the *Hoxd* gene cluster and genes located nearby (bottom), together with the whole-mount in situ hybridization (WISH) pattern of the five most posterior *Hoxd* genes (*Hoxd9* to *Hoxd13*) (top). The scheme illustrates both the similarity in the expression patterns and the progressive gain of expression, which correlates with the proximity of the target genes to the digit enhancers. *Evx2* and *Lnp* are co-expressed with *Hoxd* genes in the developing digits, under the control of the same regulatory elements. **b** Fluorescent in situ hybridization of *Hoxd13* mRNA on section reveals discrete expression pattern in the autopod of E12.5 mouse forelimb*.* **c** Single-cell double labelling of *Hoxd13* and *Hoxd11* mRNA from E12.5 autopod cells (up, schematic) by fluorescent hybridization followed by flow cytometry detection. The density plot (below) shows a high proportion of double negative cells. **d** *Hoxd11* expression pattern revealed using a *Hoxd11::GFP* knock-in mouse strain, with high expression in digits and low expression in interdigital cells of autopod forelimbs, together with strong signals in zeugopod cells. **e**, **f**. Scatterplot profile from FACS to enrich for *Hoxd11-*positive cells using cells with high levels of GFP fluorescence (**e**, red) subsequently double-labelled for both *Hoxd13* and *Hoxd11* mRNAs. **e** Flow cytometry analysis from the GFP-positive cells from **e** are shown in a density contour plots where colors highlight the four population of cells expressing various levels of *Hoxd11* and *Hoxd13*. **g**–**i** Double labelling of GFP (green, marker of *Hoxd11*-positive cells, **g**, **h**) and *Hoxd13* (**i**, red, FISH) with DAPI (magenta) suggests four different combinations of *Hoxd*-positive cells: double positive for *Hoxd13* and *Hoxd11*, single *Hoxd13*-positive, single *Hoxd11*-positive, and double negative for *Hoxd13* and *Hoxd11.* **j** Histograms showing the correlation of expression where high levels of *Hoxd13* are associated with high levels of *Hoxd11* (two panels on the left). Higher levels of GFP are also observed in cells expressing at least one of the *Hoxd* genes (central panel). **k** Schematic of the four different combinations observed in **f** and **h**, **i**: double negative for *Hoxd13* and *Hoxd11* (purple), single *Hoxd11*-positive (green), single *Hoxd13*-positive (red), and double positive *Hoxd13* and *Hoxd11* (yellow)

Limb morphogenesis is controlled by several key transcription factors, amongst them members of the *Hox* gene family, in particular genes from the *HoxA* and *HoxD* clusters. During early limb development, the posterior *Hoxd* genes are expressed in precise, partly overlapping domains [1], which will pre-figure the various parts of the future appendices, i.e., the hands and feet (autopods) and the more proximally located arm (stylopod) and forearm (zeugopod) segments. Recently, it was shown that the expression of five genes (from *Hoxd9* to *Hoxd13*) in presumptive digits is under the control of the same set of enhancer elements, located in the gene desert centromeric to the cluster itself [2–4] (Fig. 1a). However, their global expression patterns display some differences, with a broader expression of *Hoxd13* within the presumptive digit 1 (the thumb), whereas *Hoxd9* to *Hoxd12* transcripts were found only in presumptive digits 2 to 5 (Fig. 1a). This difference is likely due to the existence of a quantitative collinearity [5, 6], whereby a gradual increase in the amount of steady-state mRNA levels is observed from *Hoxd9*, expressed at the weakest level, to the robust transcription of *Hoxd13*, the latter being located on the side of the corresponding enhancers.

Chromatin interactions between *Hox* genes and their enhancers in single cells showed variability [4, 7, 8], and super-resolution microscopy confirmed that the *HoxD* gene cluster can display a variety of structural conformations in future autopod cells [9]. This heterogeneity is difficult to integrate with chromosome conformation datasets produced at this locus, since the latter approach reflects the averaged behaviors of a cellular population. Consequently, a higher variability can be expected in cell-specific *Hox* gene transcriptions, when compared to the apparently rather homogenous expression profiles previously reported [6, 8].

Moreover, while genetic approaches have revealed the critical function of these genes during limb outgrowth and patterning, the homogeneous or heterogeneous impact of mutations at the cellular level is more difficult to evaluate. The ablation of *Hoxd13* alone leads to a morphological effect in digits weaker than when a simultaneous deletion of *Hoxd11*, *Hoxd12*, and *Hoxd13* is achieved [10–12], suggesting that *Hoxd11*, *Hoxd12*, and *Hoxd13* functionally cooperate during digit development. However, how this cooperation occurs at the cellular level is unknown.

One potential cause for transcriptional heterogeneity may involve a competition between the various promoters located *in-cis* and the global enhancers driving transcription in digits [13]. It was indeed recently reported that the *HoxD* cluster lies between two large topologically associating domains (TADs) [14, 15] each of them containing series of enhancer elements with distinct specificities [3, 7]. The TAD located centromeric to *HoxD* (C-DOM) contains several enhancers specific for autopod (digit) cells, whereas T-DOM, the TAD located telomeric to *HoxD*, hosts a series of enhancers specific for the future arm and forearm cells. While genes located at either extremity of the cluster respond to their neighboring TAD, those genes located at a central position in the cluster such as *Hoxd9*, *Hoxd10*, or *Hoxd11* are targeted successively by enhancers belonging to the two different TADs. Initially, in future forearm cells, they respond to T-DOM regulation, whereas in a subsequent phase, in future digit cells, they respond to C-DOM enhancers [16], which may lead to an even greater heterogeneity in transcript distribution. In order to try and evaluate the heterogeneity in *Hoxd* transcript distribution during limb development, we produced single-limb cell transcriptomes of different origins, to see whether the apparently homogenous *Hox* gene transcriptional program as observed upon large-scale analyses could be observed at the cellular level as well. Here, we report that *Hoxd* gene transcripts are present in various combinations in different limb cells. We discuss the impact of these results upon our understanding of how *Hoxd* genes are regulated and how their global functions are achieved in these structures.

## Results

### Heterogenous distribution of posterior *Hoxd* gene transcripts in single cells

In order to document the expression pattern of *Hoxd13* at the single-cell level, embryonic day (E) 12.5 limb sections were use in RNA-FISH experiments (Fig. 1b). As expected, we observed a high expression specificity in presumptive digit cells in the distal part of the forelimb, with the highest transcript levels in cells located at the boundary between the digital and the interdigital compartments, while lower levels were scored in interdigital mesenchyme. Signal was detected neither within the digital compartment nor in more proximal parts of the limb [6] (Fig. 1b). However, a high heterogeneity in gene expression was recorded, with stippled signal pattern contrasting with the broader expression domain previously described by whole-mount in situ hybridization (WISH). As a consequence, we asked whether all cells expressing *Hoxd13* would also contain *Hoxd11* transcripts, knowing that both genes are under the same regulatory control in these distal cells [2, 17]. We micro-dissected autopod tissue to obtain a single-cell suspension and performed double fluorescent RNA labelling. The single-cell preparation was then analyzed by fluorescence-activated cell sorting (FACS) and revealed that only a minority of cells was in fact expressing *Hoxd11* and/or *Hoxd13* (Fig. 1c). Amongst positive cells, the largest fraction was *Hoxd13* positive and negative for *Hoxd11* (*d13*^*+*^*d11*^*−*^; 53%), whereas double positive cells (*d13*^*+*^*d11*^*+*^) represented 38% only and 9% of the cells contained *Hoxd11* mRNAs alone (*d11*^*+*^) (Fig. 1c).

Because a substantial number of cells did not express any *Hoxd* genes, we enriched for the positive fraction using a mouse line containing a GFP reporter sequence knocked in *Hoxd11*. In these mice, GFP was produced in those cells where *Hoxd11* had been transcribed (Additional file 1: Fig. S1). We monitored the fluorescence at E12.5 and observed a pattern recapitulating *Hoxd11* endogenous expression (Fig. 1d). E12.5 limb cells from these animals were FACS-sorted using the GFP (Fig. 1e) and, under these conditions, the double labelling of GFP-positive cells increased to more than a third of the cells (Fig. 1f). However, amongst the positive cells, the ratio between the three *Hoxd*-positive populations (*Hoxd13* only, *Hoxd11* only, and double positive) was roughly the same as before (37%, 7%, and 55%, respectively). To confirm the presence of these different populations, we performed *Hoxd13* RNA-FISH on sections from *Hoxd11::GFP* E12.5 forelimbs (Fig. 1g) and observed a high variability in GFP levels (Fig. 1h). We found that high levels of *Hoxd13* were observed in cells with either little or no *Hoxd11* activity (Fig. 1i), yet the majority of cells displayed high signals for both *Hoxd11* and *Hoxd13*, suggesting that in these cells the two genes were regulated in a similar manner.

To quantify a potential correlation between *Hoxd11* and *Hoxd13* expression levels in these GFP-positive cells, we binned *Hoxd11*-positive cells in three categories: negative cells (*d11neg*, orange), cells expressing at low levels (*d11low*, red), and cells expressing at high levels (*d11hi*, gray; Fig. 1j, left panel). Flow cytometry analysis revealed that higher *Hoxd13* levels were clearly observed in the *d11hi* population, indicating that in single cells, whenever both genes are expressed, they tend to respond to enhancers with the same efficiency (Fig. 1j). To relate these latter results with the level of GFP observed by microscopy (Fig. 1i), we monitored the levels of GFP in single cells and found a correlation between abundant *Hoxd11* mRNAs, on the one hand, and higher levels of the GFP protein, on the other hand (Fig. 1j, right panel). Altogether, these results suggested that some cellular heterogeneity exists with respect to *Hoxd* gene transcription in presumptive digit cells, with the possibility for sub-populations of cells to selectively express either one or two genes. Overall, these observations contrasted with the view that all limb cells transcribe all posterior *Hoxd* genes, a view conveyed by the global analysis of expression patterns by whole-mount in situ hybridization (WISH) and accentuated by schematics published to summarize these expression domains (e.g., [18]).

### Single-limb cell transcriptomics

To have a wider view of this cellular heterogeneity by expanding the analysis to all *Hox* genes, as well as to see whether it depends on the position and fate of various limb cells, we performed single-cell RNA-seq. Because of its potential to detect as little as single-digit input spike-in molecules, we used the Fluidigm microfluidics C1 captures to obtain the maximal intensity of transcript detection [19]. We enriched for cells expressing at least one *Hoxd* gene by using only the GFP-positive cells sorted by flow cytometry from the *Hoxd1I::GFP* mouse E12.5 forelimbs (see Fig. 1d–f). After capture, the cells were sequenced at very high depth to reach the finest sensitivity of gene detection, with an average of about 8.7 M reads per cell (Additional file 2: Fig. S2).

**Fig. 2:**
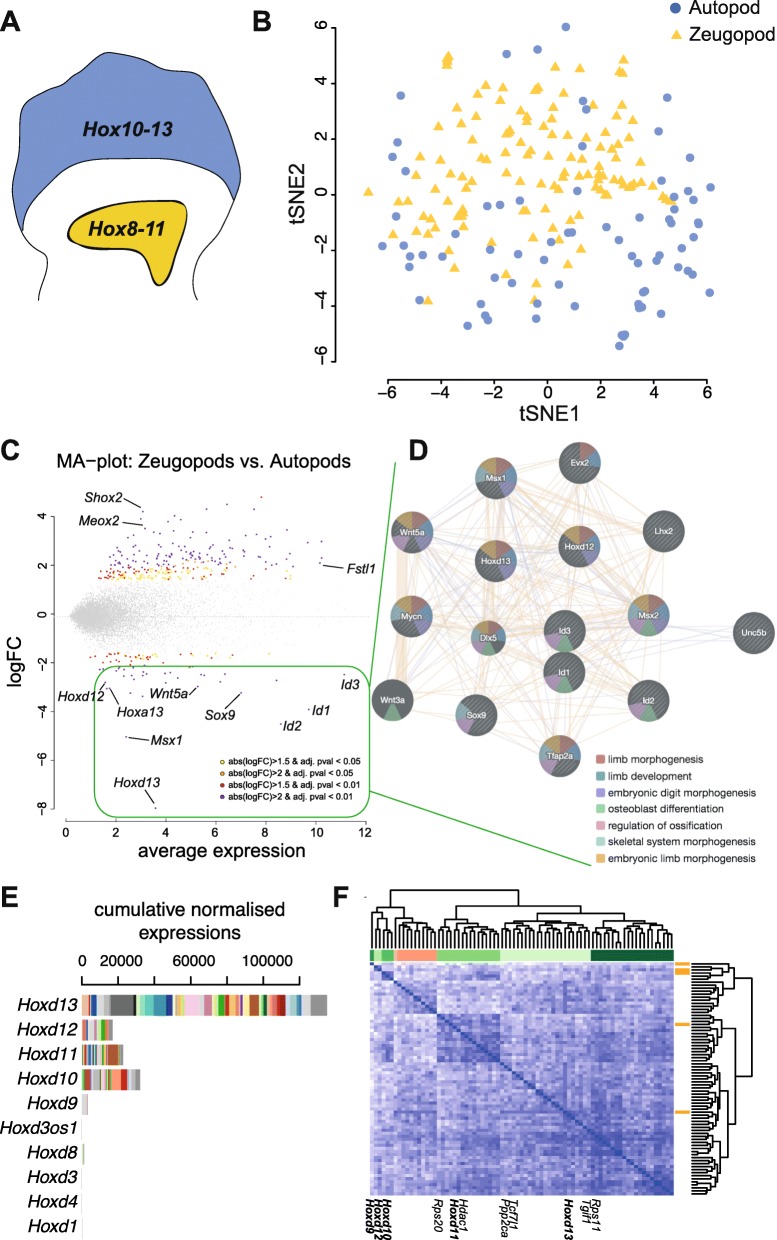
Single-cell transcriptomics from forelimb autopod and zeugopod. **a** Schematics of forelimb territories harboring different combinations of *Hoxd* gene activity. Single-cell RNA-seq was performed on micro-dissected 12.5 forelimb autopod (AP, blue) and zeugopod (ZP, yellow) tissues derived from *Hoxd11::GFP* limbs and positive for GFP. **b** T-distributed stochastic neighbor embedding (tSNE) plot of gene expression relationships amongst the 199 single cells from AP (blue) and ZP (yellow) shows a segregation along the tSNE2 axis. **c** MA plot produced from cross-analysis between AP and ZP cells. Known genes with differential expression between tissues are indicated on the graph. **d** Gene nodes from the differentially expressed genes (DEG) upregulated in autopod tissue, as computed using co-expression and interaction meta-analysis. **e** Cumulative combinatorial expression of *Hoxd* genes in the autopod cells indicating variability of gene expression across *Hoxd* genes from cell to cell. **f** Spearman’s rank correlation heatmap and hierarchical clustering of genes that covaried with at least one posterior *Hoxd* gene in the autopod cells. Below are shown the names of *Hoxd* genes (bold) and their known target genes

The analysis of these transcriptomes showed that autopod and zeugopod cells portray distinct transcriptional signatures, as observed with a machine learning algorithm that reduces dimensionality (t-SNE). In this plot representation, we saw little intermingling only between autopod and zeugopod cells (Fig. 2a, b). To ensure that the single-cell signatures were specific to the two populations, we performed a differential expression analysis between the distal and proximal limbs. As shown in the MA plot, we found that genes specific to one or the other populations were indeed known markers of the two tissues (Fig. 2c and Additional file 3: Table S1). In fact, most of the autopod-specific genes are part of a tight interactive network established through weighted aggregation of known interactions (Fig. 2d and Additional file 4: Fig. S3), thus confirming the high level of gene detection in our single cells (Fig. 2d and Additional file 2: Fig. S2).

To visualize the relative mRNA contributions from all *Hoxd* genes, we plotted their cumulative expressions with color-coded single cell (Fig. 2e and Additional file 5: Fig. S4). While the distribution of absolute levels mirrored quite well the pattern previously established using other approaches [4, 6], we observed again a selectivity in expression, which also applied to *Hoxd12*, *Hoxd11*, and *Hoxd10*. Of note, amongst autopod cells positive either for *Hoxd13* and/or for *Hoxd11*, we identified similar proportions as before, with the majority of cells expressing both *Hoxd11* and *Hoxd13*, 40% containing *Hoxd13* mRNAs only and 10% with *Hoxd11* mRNAs only.

To assess the potential covariances between the five *Hoxd* genes important for limb development (from *Hoxd9* to *Hoxd13*), we classified by Spearman’s rank correlation the genes that covaried with at least one of the *Hoxd* genes. A hierarchical clustering from these 76 genes showed a clear segregation between *Hoxd11/Hoxd13*, on the one hand, and *Hoxd9*, *Hoxd10*, and *Hoxd12*, on the other hand (Fig. 2f). While *Hoxd9*, *Hoxd10*, and *Hoxd12* were closely associated in either the presence or the absence of their mRNAs, *Hoxd11* and *Hoxd13* were part of two different sub-clusters associated with different set of genes, suggesting that the cell-specific expression of combinations of *Hoxd* genes may have some biological relevance.

### Combinatorial *Hoxd* gene expression observed in single limb bud cells

**Fig. 3:**
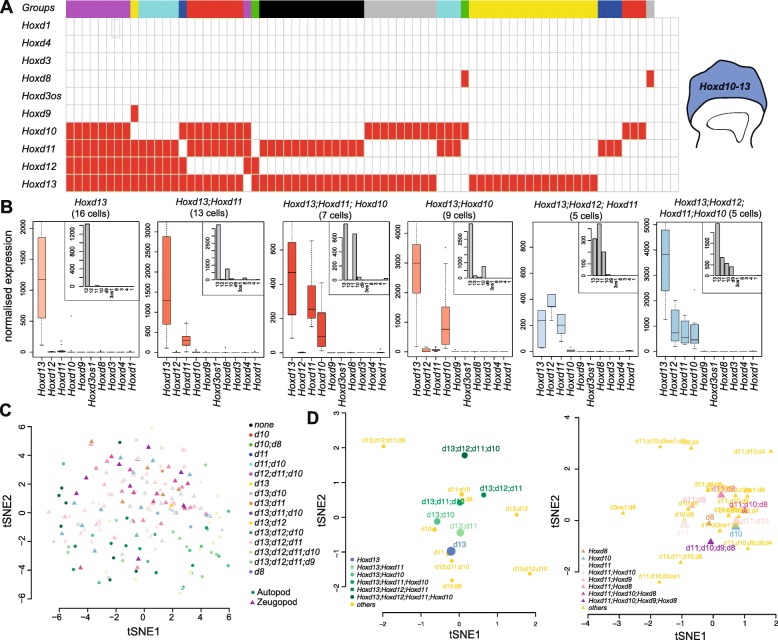
Combinatorial *Hoxd* gene expression in single cells. **a** Supervised cluster analysis reveals 16 combinations (clusters) of posterior *Hoxd* genes in autopod single cells. **b** Boxplots showing normalized expression for all *Hoxd* genes for the six main clusters from autopod cells, with a representative single cell shown in the top-right corner. **c** tSNE with color-coded combinations of posterior *Hoxd* genes in autopods and zeugopods. **d** tSNE showing groups of cells sharing the same combination of expression in autopod (left) and zeugopod (right)

Therefore, despite their shared tissue-specific regulatory landscapes, *Hoxd* genes are not systematically expressed together in the same cells. A discretization of the expression levels allowed us to score the various mRNA combinations observed either in autopod (Fig. 3a) or in zeugopod (Additional file 6: Fig. S5) single cells. In the autopod, the largest population was composed of cells expressing *Hoxd13* only, followed by a population expressing both *Hoxd11* and *Hoxd13* and then by an unexpected pool of cells with only *Hoxd10* and *Hoxd13* mRNAs. Cells containing three or more distinct *Hoxd* mRNAs were a minority and only 11% of cells expressed four genes, from *Hoxd10* to *Hoxd13*. We asked whether these unambiguous associations were random or coupled with specific gene signatures by performing a T-distributed stochastic neighbor embedding (tSNE) on all autopod and zeugopod cells. We observed that groups of cells containing different combinations of *Hoxd* mRNAs tend to segregate, suggesting that their differences in gene expression is not restricted to *Hoxd* genes only (Fig. 3c).

We then performed separate tSNE for autopod and zeugopod cells by clustering cells according to their *Hoxd* combinatorial patterns (Fig. 3d) and observed that some combinations tend to cluster together. This effect was particularly clear in autopod cells whenever a sufficient number of cells (> 5) were plotted and we noticed that the transcriptional diversity increased along the second dimension of the tSNE, when a higher diversity of *Hoxd* mRNAs was scored in the same cells. In zeugopod cells, groups of cells also segregated, though not as distinctly, suggesting a more homogeneous distribution of *Hoxd* mRNAs. These results suggested that sub-populations of autopod cells transcribe various combinations of *Hoxd* genes.

### Analysis of *Hoxd* cellular clusters

**Fig. 4:**
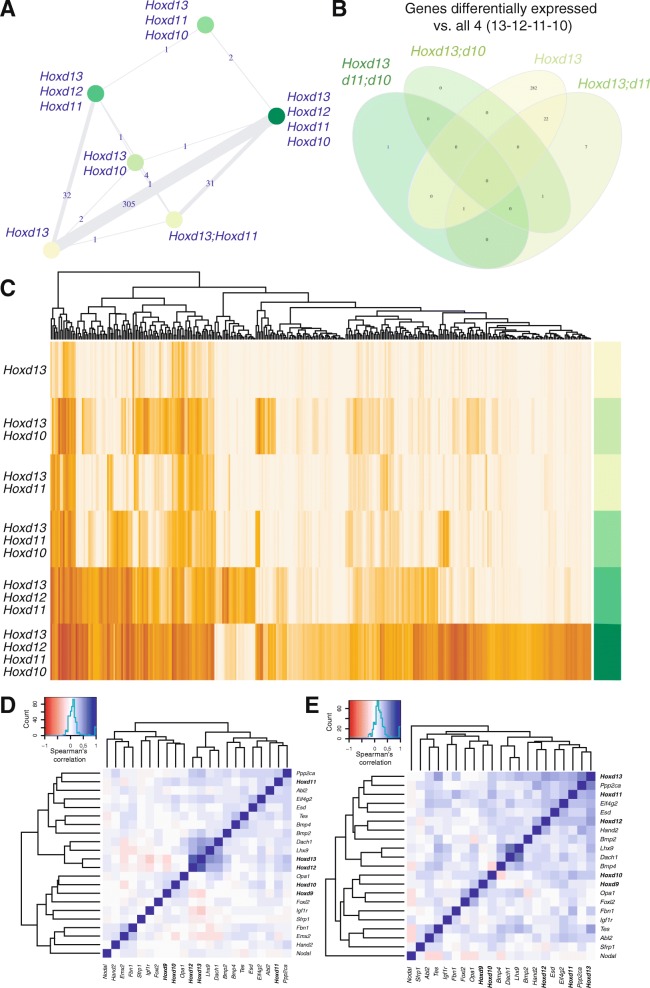
Analysis of *Hoxd* mRNA combinations clusters (**a**). Network diagram of differentially expressed genes between the six main combinations of posterior *Hoxd* genes. **b** Venn diagram showing the number of overlapping genes differentially expressed between the same combinations. **c** Heatmap and unsupervised clustering of the 343 differentially expressed genes in the six groups of cells. **d**, **e** Spearman’s rank correlation heatmaps and clustering of posterior *Hoxd* genes and their targets in the full set of 199 cells from autopod and zeugopod (**d**) and only in autopod cells (**e**)

To more precisely assess this apparent cellular selectivity in *Hoxd* gene expression, we determined whether particular cell clusters were at a specific phase of the cell cycle. While most cells with G2 scores were observed either with *Hoxd13* mRNAs only or with four posterior *Hoxd* genes active, we did not detect any significant difference associated with a specific combination of mRNAs (Additional file 6: Fig. S5). We next performed a differential gene expression analysis to assess the degree of relationship between the six main cellular groups (Fig. 4a–c). Most of the differentially expressed genes (343 genes, Additional file 7: Table S2 and Additional file 8: Fig. S6) were scored between cells expressing only *Hoxd13* and cells expressing either three (*Hoxd11*–*Hoxd13*) or four (*Hoxd10* to *Hoxd13*) genes (Fig. 4a). Amongst these differentially expressed genes, many displayed strong autopod expression, including *Jag1*, which is downregulated in the absence of the HOX13 proteins [20]. Out of 31 genes differentially expressed between cells containing either *Hoxd13* and *Hoxd11* mRNAs or *Hoxd10*, *Hoxd11*, *Hoxd12*, and *Hoxd13* mRNAs, only eight were specific to these two combinations, i.e., *Smarcc1*, *Mrps17*, *Snrpd2*, *Supt6*, *Tax1bp1*, *Rab5c*, *Ncbp2*, and *Map3k7*.

Noteworthy, the clustering of expressed transcripts showed a hierarchical organization with a progression from those cells expressing *Hoxd13* only to two, then three, and finally four *Hoxd* genes (Fig. 4c). As some of these genes were previously identified either as HOX protein targets (e.g., *Ppp2ca* [21]) or being part of a *Hox* functional pathways (e.g., *Uty* [22, 23] or *Hoxa11os* [24, 25]), we assessed whether specific target genes could be associated with particular combinations of *Hoxd* mRNAs. We generated a supervised clustering showing the covariance of known target genes in a Spearman correlation matrix (Fig. 4d, e). When the 199 cells originating from both the autopod and the zeugopod were considered, we found a clear partition of target gene mRNAs into two groups corresponding to the nature of *Hoxd* mRNAs present (Fig. 4d). The presence of *Hoxd9* and *Hoxd10* mRNAs aggregated with targets genes such as *Hand2* and *Sfrp1*, whereas *Hoxd11*, *Hoxd12*, and *Hoxd13* were co-expressed with different target genes such as *Ppp2ca* and *Bmp2/4*. Finally, the highest clustering across all cells was observed between *Hoxd12*, *Hoxd13*, *Dach1*, and *Lhx9*, thus revealing a robust link between these genes.

When only autopod cells were considered, we observed two groups, with *Hoxd9* and *Hoxd10* transcripts in one cluster, while the more centromeric genes *Hoxd11*, *Hoxd12*, and *Hoxd13* were transcribed in the other (Fig. 4e). As the former group did not express any of those genes typically upregulated in distal cells (*Hoxd12* and *Hoxd13*), we wondered whether such differences in transcript distribution may reflect various stages in the progression of distal limb cells towards their final fates. We thus implemented a measure of cellular pseudo-age, a strategy that evaluates a temporal hierarchy amongst single cells based on their respective transcriptomes. This approach allows to plot cells along a linearized axis to infer whether the combination alignments observed in the tSNE may correlate with a modulation of the time component [26–28].

**Fig. 5:**
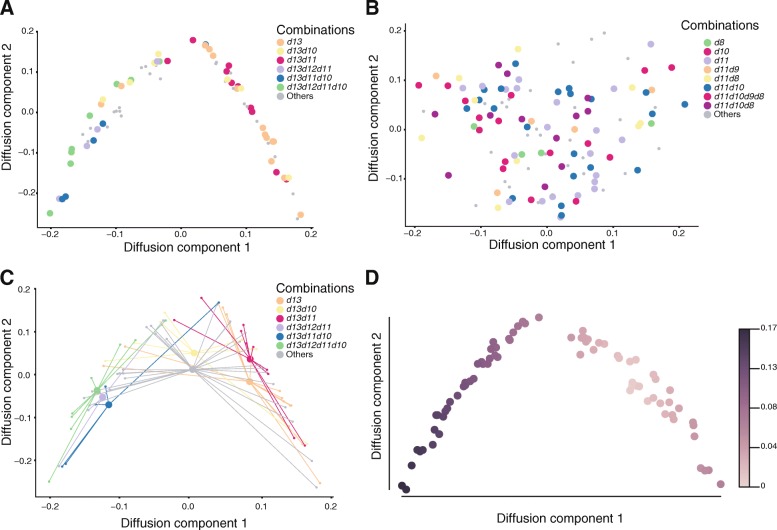
Diffusion pseudo-time across single cells. **a** Diffusion maps of the 76 autopod cells colored per combinations of *Hoxd* mRNAs. This dot plot shows a progression from the simplest combinations to more complex ones. The most significant dimensions, i.e., the first two eigenvectors (DC1 and DC2) are displayed. **b** Diffusion maps of the 120 zeugopod cells colored per *Hoxd* mRNA combinations. **c** *Hoxd* groups centroid. The centroid of a *Hoxd* group, represented in the diffusion map of the 76 autopod cells. **d** Distribution of autopod cells as shown in **a** are color-coded to show their progression along diffusion pseudo-time

We performed such a pseudo-time analysis on the single cells isolated from both the autopod and zeugopod and found that cells indeed spread along the pseudo-temporal axis that was linearized through a diffusion map (Fig. 5a, b). This was also the case when we plotted cells originating from a single female embryo, which illustrates that the maturation is not due to cells coming from embryos at slightly different developmental stages (Additional file 9: Fig. S7). In these maps, while zeugopod cells did not distribute well along a temporal frame (Fig. 5b), the autopod cells were much better aligned (Fig. 5a). As illustrated with gene expression clustering (Fig. 4a–c), specific combinations are distributed along the temporal axis in a way related to the various combinations of *Hoxd* mRNAs, with the *Hoxd13*-only cells at one extremity of the axis and the *Hoxd10* to *Hoxd13* combination at the other extremity (Fig. 5c, d). Altogether, this clustering analysis showed that different combinations of *Hoxd* gene mRNAs may affect distinct groups of target genes. Of note, it also revealed a preference for mRNA combinations involving neighbor genes, thus emphasizing the importance of genes’ respective positions for their co-regulation.

## Discussion

During the early stages of limb growth and patterning, limb bud cells absolutely require the expression of *Hox* genes originating from two distinct clusters, *HoxA* and *HoxD* [29, 30]. Here, we describe the single-cell combinatorial expression of *Hoxd* genes found in cells sorted out by using a *Hoxd11*::GFP mouse strain. Albeit some cells tend to show higher level of *Hoxa* genes whenever the levels of *Hoxd* transcripts were low, this was not the general rule. The fact that we did not score many *Hoxa* mRNA-positive cells after the enrichment for *Hoxd* gene expression (Additional file 5: Fig. S4) may however reflect a compensatory mechanism whereby a strong global expression of one cluster would result in the weak transcription of the other. Distinct cellular content for either *Hoxd* or *Hoxa* mRNAs could account for the different phenotypic effects of inactivating these genes upon limb morphology, as exemplified by *Hoxd13* and *Hoxa13* [18, 29, 30]. A more comprehensive single-limb cell sequencing strategy will likely fix this issue.

Our data show that *Hoxd* quantitative (or “reverse”) collinearity [5, 6] [31] ought to be considered at a global level since it results in fact from a sum of combinatorial expression of various genes in different cells. We emphasized that in autopod cells, the most frequently expressed gene is *Hoxd13*, as was expected from previous studies where it was described that this gene is expressed at the highest level, due to its position at the extremity of the gene cluster, i.e., on the centromeric side of a strong TAD boundary [6, 7]. Apart from *Hoxd13*, other *Hoxd* genes were more sparsely activated, indicating either a stochastic process or a functional requirement for specific mRNA combinations in different cell types. This heterogeneous cellular situation raises two separate questions; the first concerns the underlying regulatory mechanism, whereas the second has to do with a potential functional significance of these different mRNA combinations.

### Different regulatory conformations?

We had previously shown that the regulation of posterior *Hoxd* genes in the distal limb bud was not implemented exactly in the same manner for all genes. In particular, *Hoxd13* is the only gene to be expressed in presumptive thumb cells, the other *Hoxd* mRNAs being excluded from this very digit [6, 32]. Also, the deletion of the *Hoxd13* locus lead to the upregulation of *Hoxd12* in thumb cells, yet not of the other remaining genes, suggesting that this thumb-specific expression was associated with the final and most 5′position of the gene on the cluster [13]. The recent identification, in the posterior part of the *HoxD* cluster, of an unusually high density of bound CTCF molecules may cause this transcriptional selectivity through the use of various sites, taking advantage of their distinct orientations [4, 7, 33].

In this view, the particular orientations of CTCF-binding sites may allow for the transient stabilization of various loop conformations, for example after extrusion driven by the cohesin complex [34, 35]. Accordingly, distinct combinations of posterior *Hoxd* mRNAs could reflect the formation of specific loop extrusion patterns, in any single cell, as a choice between a fixed number of possibilities determined by the presence of bound CTCF, with some conformations being favored over others. Of note, we found that the cohesin-loading factor *Nipbl* is strongly downregulated in cells from the *Hoxd13* group when compared to cells expressing the full combination (*Hoxd10* to *Hoxd13*). Mutations in this gene have been found in patients with Cornelia de Lange syndrome who have notably a clinodactyly of the fifth finger. Also, a recent report showed that mice heterozygotes for *Nipbl* display polydactyly and that lower dose of *Hoxd11* to *Hoxd13* in these mice could further enhance this phenotype [36].

Other chromatin regulators were found enriched in this list of genes including *Jag1*, *Brd7*, *Jmjd6*, *Phf8*, *Ddb1*, *Hdac1*, *Swi5*, *Smarcc1*, *Smarce1*, *Hmgb3*, *Dnm3os*, *Cbx1*, and *Lmnb1* (Additional file 3: Table S1). The product of the latter gene has been associated with the architecture of large domains of inactive chromatin (LADs; [37]), where the *HoxD* cluster is not located [38]. Since reduced levels of *Lmnb1* gene product have been shown to be associated with reduced expression of polycomb target genes, including the posterior *Hoxd* genes [39], its increased expression in those cells containing mRNAs from *Hoxd10* to *Hoxd13* may reflect a global change in chromatin configuration [37, 40–42]. How would this change relate to a more permissive expression of *Hoxd* genes, as a cause or as a consequence, remains to be established.

The analysis of single-cell transcriptomes revealed an unexpected hierarchical progression of *Hoxd* gene expression, from cells expressing a single posterior gene (*Hoxd13*) to the full combination, from *Hoxd10* to *Hoxd13*. This global transcriptional sequence was inferred from a pseudo-time approach, a method whereby a temporal progression of cells is deduced based on their transcript patterns [26, 28]. We tested this hypothesis using diffusion pseudo-time and found that autopod cells are much more subject to align along a developmental trajectory. This specificity may be associated to the particular way *Hoxd* genes are regulated in distal limb buds, with a rapid and strong activation of *Hoxd13* due to its leading position in the cluster favoring privileged contacts with the various enhancers [2, 43]. It is possible that the recruitment of additional *Hoxd* genes located nearby may be more progressive, along with local epigenetic modifications, which could be inherited from one cell to its daughter cells. In this view, the number of *Hoxd* genes expressed would increase along with mitotic divisions leading to the hierarchical progression observed.

### Additive cellular or emerging functions?

The second question relates to the potential different functions that limb bud cells may display by carrying distinct combinations of *Hoxd* mRNAs. The question here is to discriminate between two views of the genotype-phenotype relationship during limb bud development; in a first scenario, each cell would express a determined combination of *Hoxd* mRNAs, for example in response either to its topological position within the growing limb or to its own “regulatory history,” i.e., the regulations at work in its ancestor cells. In a second scenario, a balanced distribution of cells expressing various *Hoxd* mRNAs could result from a stochastic distribution of a fixed set of various chromatin architectures [44]. In the former context, the resulting limb phenotype would derive from the additive effect of every single cell, providing one out of the possible sets of information delivered by the various transcriptomes associated. In the second framework, the phenotype would derive from the random mixture of multiple cells expressing distinct transcriptomes with a given balance fixed by the choice of one possible chromatin conformations. Under physiological conditions, these various combinations may allow for differential responses to signaling molecules to generate cellular diversity at the time digit patterns are being established across the limb. It remains to be seen how such potential modulations in the responses to signaling pathways may integrate a theoretical framework whereby digit formation may be an emerging property of the this cellular system [45, 46].

Genetic approaches cannot easily discriminate between these alternatives. In previous studies where the functions of *Hoxd* genes during limb development were aimed to be assessed separately, various combinations of multiple gene inactivation were used. In most cases however, this consistently led to limited phenotypes due to a fair level of redundancy, particularly amongst *Hoxd* and *Hoxa* genes, preventing precise functions to be attributed to specific (groups of) *Hox* genes (see refs in [18]). However, the use of multiple gene inactivation revealed that the transcription of *Hoxd11* and *Hoxd12* contributed functionally and thus added to the mere presence of *Hoxd13* transcripts, even though autopods double mutant for *Hoxd13* and *Hoxa13* would no longer grow and develop digits [12, 29, 43]. This is coherent with our data suggesting that the specific presence of *Hoxd11* or *Hoxd12* mRNAs is associated with distinct transcriptomes containing additional key regulators of cell fate and chromatin remodeling genes.

Therefore, part of the limb phenotypes observed in *Hoxd* multiple mutant alleles may result from the different response of a sub-group of cells, which would be differentially impacted by the loss of a given gene. For example, cells that express only *Hoxd13* or a combination of *Hoxd13* and *Hoxd10* mRNAs may not be sensitive to the absence of *Hoxd11* transcripts in the corresponding mutant stock. Our results thus stress the necessity to keep in mind the cellular heterogeneity of transcriptional programs even in instances where WISH patterns seem to reveal homogenous distributions of transcripts. In this context, transcript patterns at the single-cell level can help solve the interpretation of genetically deficient phenotypes, even though the co-regulation of *Hoxd* genes and the functional redundancy of their products make this statement difficult to apply to the present work.

## Conclusions

Our results reveal the existence of distinct combinations of *Hoxd* genes at the single-cell level during limb development. In addition, we document that the increasing combinatorial expression of *Hoxd* genes in this tissue is associated with specific transcriptional signatures and that these signatures illustrate a time progression in the differentiation of these cells. While this cellular heterogeneity in the combinations of *Hox* mRNAs may help in understanding the complex transcriptional regulation of these neighbor genes, it will have to be considered when considering the phenotypic outcome of functional studies where one or several such genes were inactivated. Also, further analysis at different developmental stages may enable the reconstruction of the cell fate trajectories and the state transitions that causes the cellular heterogeneity of the early limb bud tissue.

## Methods

### Animal experimentation

Forelimb tissue samples were isolated from *Hoxd11::GFP* heterozygous animals at embryonic day 12.5 (E12.5) with day E0.5 being noon on the day of the vaginal plug. The cloning steps for the generation of the *Hoxd11* transgenic mice is described in (Additional file 1: Fig. S1). Briefly, the knock-in was done by introducing a bi-cistronic cassette along with an IRES sequence. *Hoxd11* was inactivated by the insertion of a *TauGFP* sequence in a frame into the coding sequence. The BamH1 site was used for insertion of the IRES cassette. The cassette was introduced as a single-copy knock-in (Additional file 1: Fig. S1). The GFP signal detected in this mouse stock reflects the endogenous distribution of *Hoxd11* transcription.

### RNA-FISH

E12.5 forelimbs were micro-dissected and fixed with 4% paraformaldehyde for 3 h. Then, the limbs were treated with sucrose at 5, 10, and 15% and then frozen in OCT. Twenty-five-micrometer cryostat sections were dried for 30 min, post-fixed in 4% paraformaldehyde for 10 min, and quenched with 0.6% H2O2 in methanol for 20 min. Slides were then processed using the Ventana Discovery xT with the RiboMap kit. The pre-treatment was performed with mild heating in CC2 for 12 min, followed by protease3 (Ventana, Roche) for 20 min at room temperature. Finally, the sections were hybridized using automated system (Ventana) with a *Hoxd13* probe diluted 1:1000 in ribohyde at 64 °C for 6 h. Three washes of 8 min in 2× SSC followed at hybridization temperature (64 °C). Slides were incubated with anti-DIG POD (Roche Diagnostics) for 1 h at 37 °C in BSA 1% followed by a 10-min revelation with TSA substrate (Perkin Elmer) and 10 min DAPI. Slides were mounted in ProLong fluorogold. Images were acquired using a B/W CCD ORCA ER B7W Hamamatsu camera associated with an inverted Olympus IX81 microscope. The image stacks with a 2-μm step were saved as TIFF stacks. Image reconstruction and deconvolution were performed using FIJI (NIH, ImageJ v1.47q) and Huygens Remote Manager (Scientific Volume Imaging, version 3.0.3).

### RNA flow cytometry

Double in situ hybridization in single cells for RNA flow cytometry was performed using PrimeFlow RNA (Affymetrix, Santa Clara, CA) reagents following the manufacturer’s protocols. Cell viability was assessed by live/dead fixable dead cell, violet (ThermoFischer; L34955). Hsp90ab RNA probe (a gene expressed ubiquitously) served as a positive control. *Hoxd11* and *Hoxd13* RNA probes were used for the actual analysis. Cell staining was analyzed on a FACS Astrios located at the EPFL flow cytometry platform. Data analysis was performed by using FlowJoX (Treestar, Ashland, OR). The labelling and flow cytometry were performed on dissociated cells from eight forelimbs obtained from four different animals pooled together.

### Single-cell dissociation and fluorescence-activated cell sorting

Pools of embryonic forelimbs obtained from eight embryos at stage E12.5 were dissociated into a single-cell suspension using collagenase from Sigma (collagenase type XI) at 37 °C for 15 min with 10 s trituration. Cells were then filtered on a cell strainer to get rid of clumps. Single cells were then resuspended in FACS solution (10% FCS in PBS with 2 mM EDTA). Fluorescence-activated cell sorting was performed using the MoFlow ASTRIOS EQ cell sorter with a 100-μm nozzle. Through flow cytometry analysis performed using FlowJo (FlowJo LLC ©), we detected 1,602,844 cells positive for GFP in the autopod tissue and 235,000 simply negative. In the zeugopod tissue, 1,527,167 cells were positive, whereas 1,296,068 were negative giving thus a total of 87% GFP-positive autopod cells and 54% positive zeugopod cells.

### Control of *Gfp* expression levels in single cells using RNAscope

To control for the differences between cells positive for the GFP protein and the absence—or low levels—of *Hoxd11*, we monitored the expression of *Gfp* mRNA using RNAscope technology (ACD, 320851). Cells were fixed and placed directly on slides following the manufacturer instructions. We used a probe against *Gfp* mRNA (#400281, C1, as designed by ACD) to assess the number of cells positive for the mRNA after being sorted, based on their fluorescence that reflects only the protein levels of GFP. Images were acquired as five Z stacks on an Axiocam (Zeiss) microscope using a 100X Plan-Neofluar × 100/1.30 Oil objective. 2D projections of the multiple planes were then transformed in mask to count *Gfp* levels per cells. Automated counting using MATLAB scored 90 positive cells for *Gfp* mRNA out of 115 cells analyzed (78%).

### Single-cell RNA sequencing, library preparation, and mapping

Dissociated single cells were obtained from eight *Hoxd11::GFP* forelimbs micro-dissected at E12.5 from four littermate embryos. Cells with the highest level of GFP fluorescence (top 20%) were sorted using an Astrios cell sorter with a 100-μm nozzle. Seventy-five-base pair large reads were uniquely mapped to the latest *Mus musculus* reference genome (mm10) and the ERCC sequences using bowtie2 [47] in a local mode. Raw counts for the annotated ENSEMBL mouse genes (GRCm38) and the ERCC were obtained using the RNA-seq module of the HTSstation portal [48]. The raw counts are summarized in (Additional file 10: Table S3). All single-cell RNA-seq data can be found in the Gene Expression Omnibus (GEO) repository under accession number GSE114748.

### Filtering low-quality cells and genes expressed at low levels

Those counts were used to filter out some low-quality cells based on the following criteria: total number of reads mapped > 250, number of genes “expressed” > 2000 (“expressed” = with count > 0), and percent of reads mapped to spike-in sequences < 25%. A total of 199 cells was retained (123 zeugopods and 76 autopods cells). As a control, the positive correlation of expression (Loess regression curve) between the *Hoxd11* and the *Gfp* RNAs is shown (Additional file 11: Fig. S8), tested by Spearman correlation with *r* = 0.69 in autopod cells and *r* = 0.49 in zeugopod cells. The *Gfp* was detected > 4 uniquely mapped reads in 84% of the cells (183 out of 199). Genes expressed at low levels were also removed from the rest of the analysis, and only genes present (raw count > 0) in at least 10% of either the 76 autopods or the 123 zeugopods cells were retained. *Hox* genes were manually added if they did not satisfy these criteria. A total of 10,948 genes remained. ERCC with null counts through the remaining cells were also excluded from the rest of the analysis. Additional file 12: Table S4 summarizes those criteria (see also Additional file 2: Fig. S2).

### Normalization

Raw counts were normalized with spike-in counts using the R package scran (methods used *computeSpikeFactors* and *normalize* version 1.0.4) (http://bioconductor.org/packages/scran/). Prior to normalization, size factors were mean-centered to their batch of origin. An additional normalization step was also applied in order to correct for a potential gene length bias. Additional file 13: Table S5 compiles all the normalized values.

### Grouping of *Hoxd* gene combinations for differential gene expression analyses

*HoxD* groups were defined per cell and were composed by *Hoxd* genes with a minimum normalized expression of 5 when count represented at least 5% of the most expressed *Hoxd* genes in the cell. The differential gene expression analysis was performed with the R package *limma* (version 3.28.21) [49]. Genes with a minimum absolute log fold change of 2 and a BH-adjusted *p* value less than 0.01 (false discovery rate (FDR) of 1%) were considered differentially expressed.

### tSNE

The tSNE were computed using the package Rtsne (version 0.13) with the following parameters: two dimensions and a perplexity of 30, a maximum of iterations of 3000, and a seed set at 42. The top highly variable genes (HVG) that were used to plot the tSNE in Fig. 3c, d were selected using the trendVar & decomposeVar methods of the R package scran (version 1.0.4) (http://bioconductor.org/packages/scran/).

### Pseudo-time

Diffusion maps [50] are tools to analyze single-cell differentiation data. It implements a distance metric relevant to how differentiation data is generated biologically, as cells follow noisy diffusion-like dynamics in the course of taking several differentiation lineage paths [26]. The distances between cells reflect the transition probability based on several paths of random walks between the cells [51]. The analysis was performed using the R package destiny (http://bioconductor.org/packages/destiny).

### Network visualization and Venn diagram

The network shown in Fig. 2 was built using weighted interaction networks from various sources of data and is able to process user data into such networks using a system that distinguishes between three different types of user-defined data in its import procedures: real- and binary-valued interaction networks, e.g., physical interaction networks; real-valued gene profile datasets, e.g., multi-sample microarray expression datasets; and binary-valued gene profile datasets [52]. The network shown in Fig. 4 is a summary network of differentially expressed genes that was made with the R package Igraph (version 1.1.2; http://igraph.org).

## Additional files

Additional file 1: **Figure S1.** Schematic of Tau::GFP targeting into the *Hoxd11* gene. The TAU::GFP sequences were introduced into the posterior part of the *HoxD* complex (A). A bi-cistronic cassette along with an IRES sequence was inserted in frame with the coding sequence of the *Hoxd11* gene (B) and a *TauGFP* (C). The BamH1 site (A) was used for the insertion of the IRES cassette. D. Schematic showing how the cassette was introduced as a single-copy knock-in. (PDF 134 kb)

Additional file 2: **Figure S2.** Concise description of the methodology and filtering methods used for the single-cell RNA-seq. A. At the top is shown a schematic depicting the Fluidigm workflow from cell dissociation to capture. Below is represented the cassette allowing the expression of GFP under the control of the *Hoxd11* endogenous promoter. The bottom left shows the GFP pattern in the E12.5 mouse embryos from which the developing limbs are dissected, sent to FACS and the cells expressing the highest level of GFP proteins captured in the C1 apparatus before libraries are built using a SMARTer kit (steps listed from left to right from left to right). B. Barplots showing the number of mapped reads per cells including the one that map on ERCC endogenous spike-ins (blue) with the number on top of each bar indicating the percentage of these ERCC amongst all reads. C. Cumulative distribution of the number of genes detected amongst all cells with the dotted lines representing the cut-off used to select only the highest qualitative cells. D. Boxplots representing the variation of the number of reads mapped per single cells with an average over 8 million reads per cells in each condition. (PDF 1562 kb)

Additional file 3: **Table S1.** List of differentially expressed genes between autopod and zeugopod cells that were sorted positive from *Hoxd11::GFP* forelimbs. Tab-delimited file. The first column indicates the genes names; all other columns represent values of average expression, fold enrichment and *p* values for each gene. (TXT 26302 kb)

Additional file 4: **Figure S3.** Table of differentially expressed genes between autopod and zeugopod cells. List of the 50 genes with the highest enrichment in autopod cells compared to zeugopod cells from E12.5 *Hoxd11::GFP*+ developing limb single cells. (PDF 26 kb)

Additional file 5: **Figure S4.** *HoxA* vs *HoxD* expression. Cumulative barplots showing *Hoxa* and *Hoxd* genes relative expression levels in autopod cells (A), zeugopod cells (B**)** and all cells together (C). (PDF 734 kb)

Additional file 6: **Figure S5.** Cyclone analysis of the cell cycle in single cells from autopod and zeugopod. A-B. Graphic representation showing the autopod (A) and zeugopod (B) cells based on their combinatorial expression of *Hoxd* genes associated with their predicted cell cycle phase as color coded with the above circles in blue (G1), yellow (G2) and green (S phase). C shows the G1 cyclone scores for each of the six main combinations in autopod cells (Right) and zeugopod cells (Left). Error bars represents standard deviation. D. Barplots showing the proportions of G1 and G2 putative state for the cells in all possible combination of posterior *Hoxd* genes (*Hoxd 9* to *Hoxd13*) observed. (PDF 201 kb)

Additional file 7: **Table S2.** List of the 343 differentially expressed genes between the six main groups of cells as shown in Fig. 4. Tab-delimited file. The first three columns indicate the coordinates of the genomic segments; all other columns represent values of individual cells. NA, no data available. (TXT 50 kb)

Additional file 8: **Figure S6.** Barplots of expression profiles for twelve representative genes that are differentially expressed between the observed combinations of posterior *Hoxd* genes in autopod cells. Top rows represent genes expressed in many combinations. Third row shows genes expressed in two or three combinations only. Bottom row shows genes only enriched in the cells expressing *Hoxd10* to *Hoxd13. (PDF 548 kb)*

Additional file 9: **Figure S7.** Single embryo analysis (related to Fig. 5). A. The distribution of autopod cells from one female embryo were plotted (black circles) along the pseudotime alignment. The others cells are shown in gray. B. *Xist* expression levels (green, left) and median expression of the top genes from the Y chromosome (purple, right) were ranked and used to filter the cells originating from one of the four embryos. Cells from this embryo (boxed at the top) are referred to as ‘Xist Rich Cells’ (XRC). (PDF 427 kb)

Additional file 10: **Table S3.** Table of the raw counts of the 225 single cells sequenced in this study. Tab-delimited file. The first three columns indicate the coordinates of the genomic segments; all other columns represent values of individual cells. NA, no data available. (TXT 11824 kb)

Additional file 11: **Figure S8.** Correlation of expression between the *Hoxd11* and *Gfp* mRNAs. The plots show for every cell the level of *Gfp* expression (X axis) and *Hoxd11* expression (Y axis), dissected either from autopod (A) or from zeugopod (B) tissue. Gene counts from all cells were used to fit a Loess regression curve (blue line) between average scaled gene counts. Pearson correlation tests are shown in the top left of each panel, with *r* = 0.69 (*p* = 5.8e^−12^; A) and *r* = 0.49 (*p* = 8.2e^−09^; B). (PDF 507 kb)

Additional file 12: **Table S4.** Table listing the values that were considering to select the cells with the highest qualities. Tab-delimited file. The first three columns indicate the coordinates of the genomic segments; all other columns represent values of individual cells. NA, no data available. (TXT 7 kb)

Additional file 13: **Table S5.** Table listing the normalized values used from the 199 selected cells used for the analysis. Tab-delimited file. The first three columns indicate the coordinates of the genomic segments. NA, no data available. (TXT 25545 kb)
