# Supplementary figures and images for: Heterogeneous combinatorial expression of Hoxd genes in single cells during limb development
Source: BMC Biol. 2018 Sep 18;16:101. doi: 10.1186/s12915-018-0570-z (PMC6142630; doi:10.1186/s12915-018-0570-z)

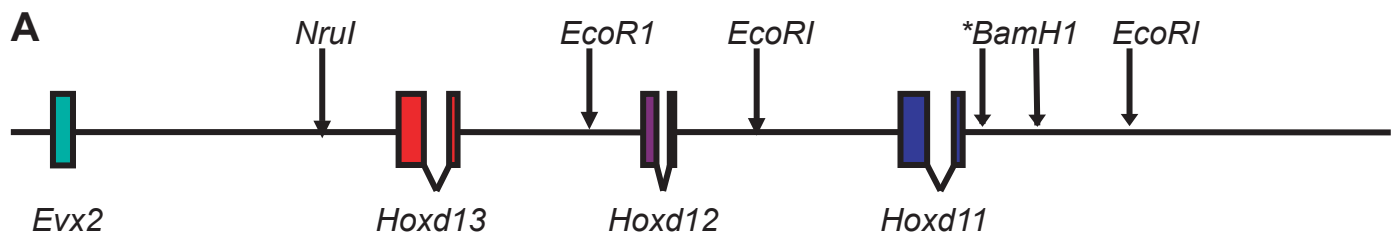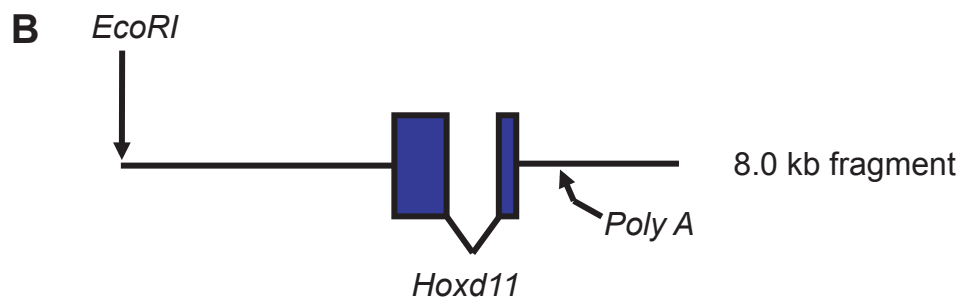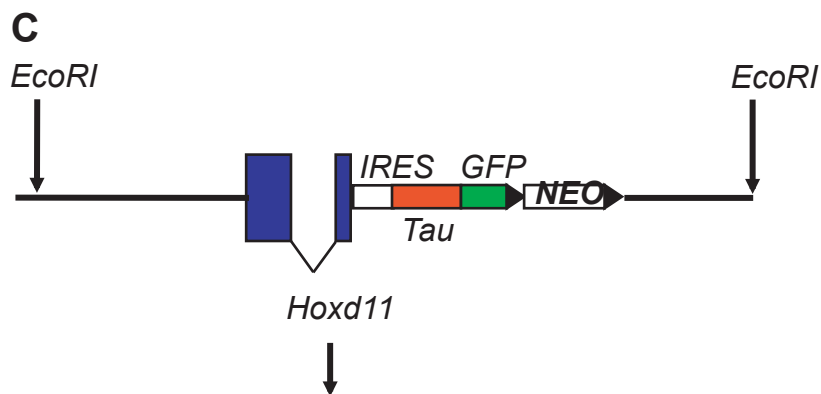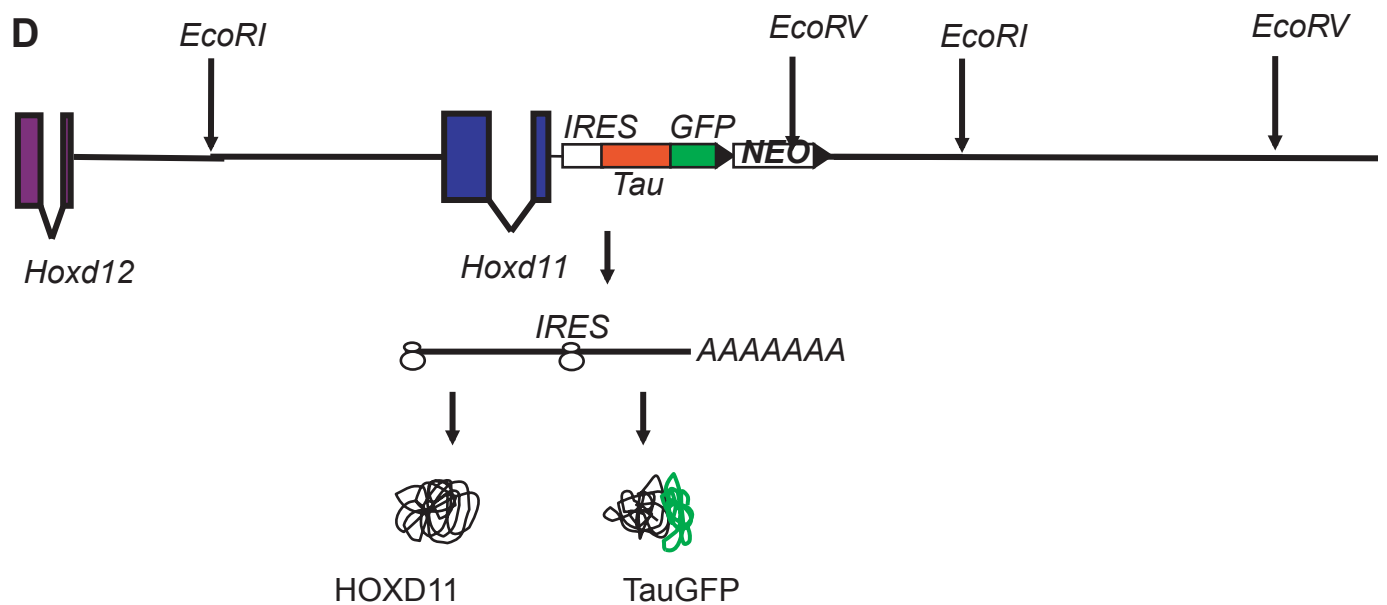

Supplement: Supplementary file 1 — Figure S1. Schematic of Tau::GFP targeting into the Hoxd11 gene. The TAU::GFP sequences were introduced into the posterior part of the HoxD complex (A). A bi-cistronic cassette along with an IRES sequence was inserted in frame with the coding sequence of the Hoxd11 gene (B) and a TauGFP (C). The BamH1 site (A) was used for the insertion of the IRES cassette. D. Schematic showing how the cassette was introduced as a single-copy knock-in. (PDF 134 kb) [file 12915_2018_570_MOESM1_ESM.pdf]

**A**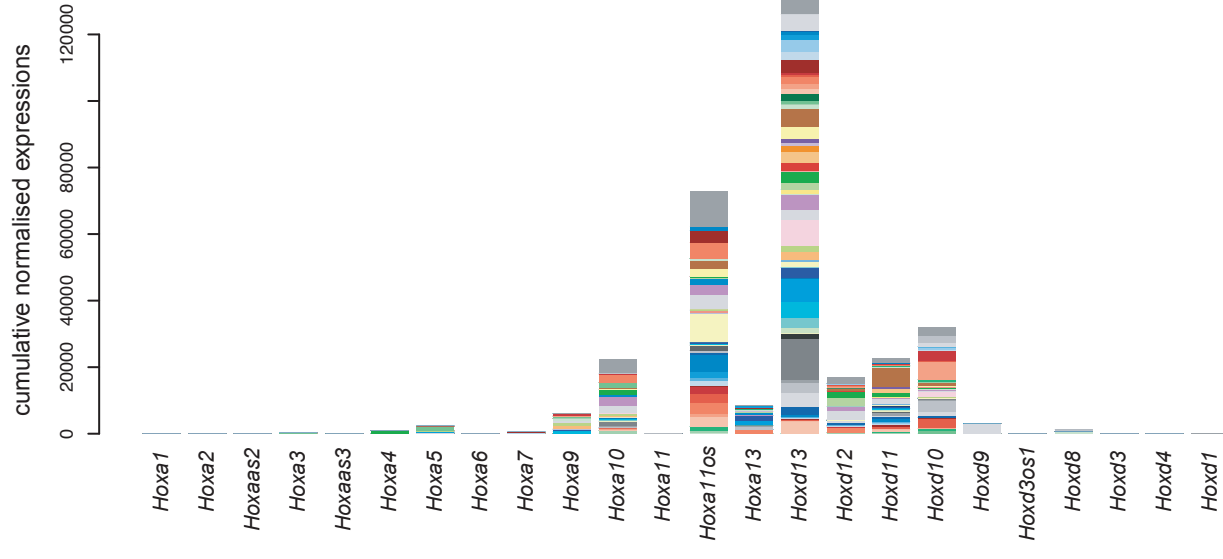**B**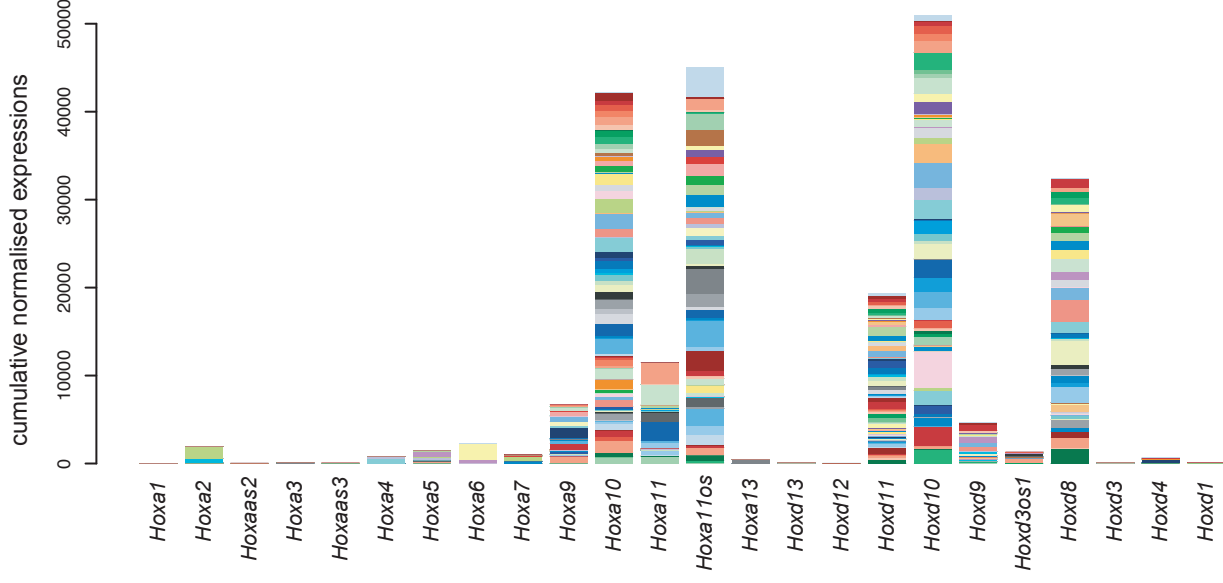**C**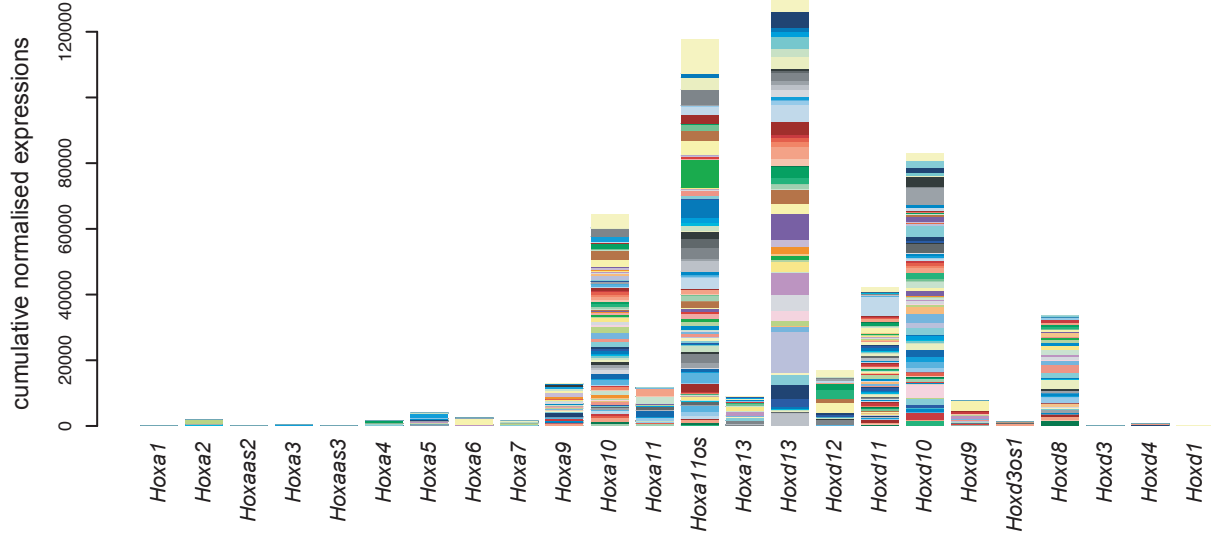

Supplement: Supplementary file 5 — Figure S4. HoxA vs HoxD expression. Cumulative barplots showing Hoxa and Hoxd genes relative expression levels in autopod cells (A), zeugopod cells (B) and all cells together (C). (PDF 734 kb) [file 12915_2018_570_MOESM5_ESM.pdf]

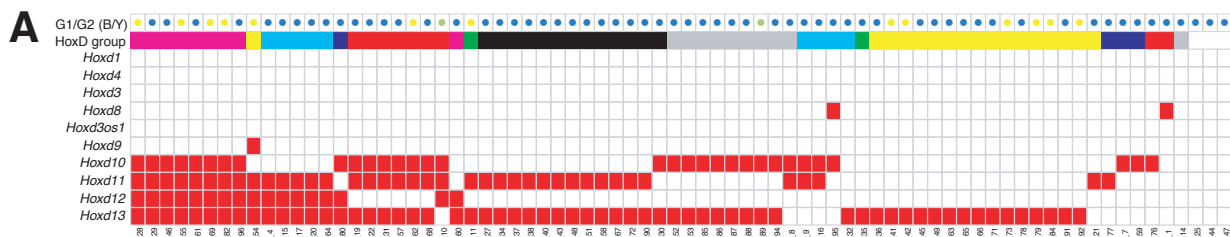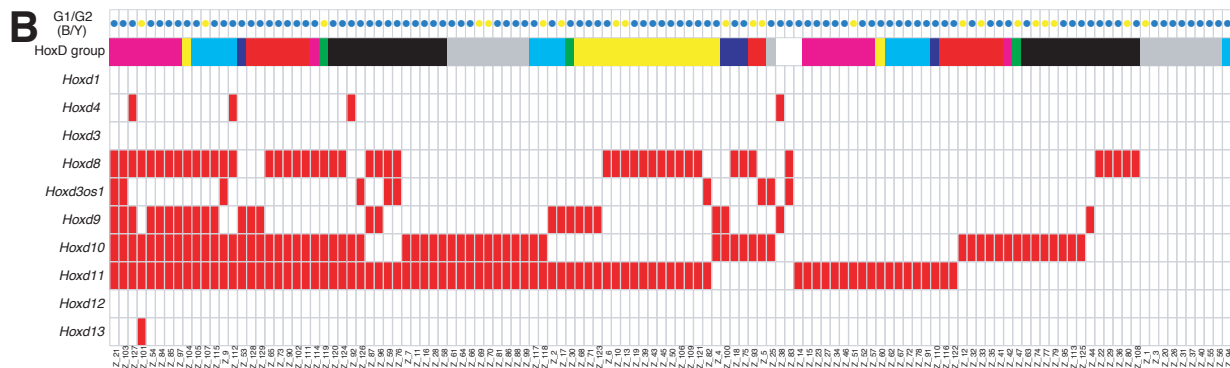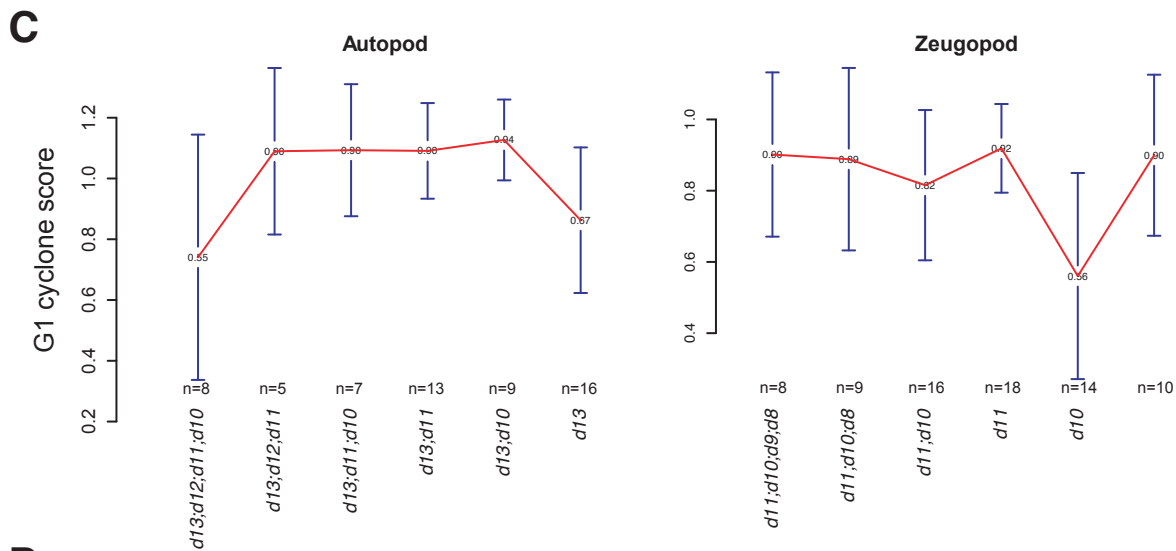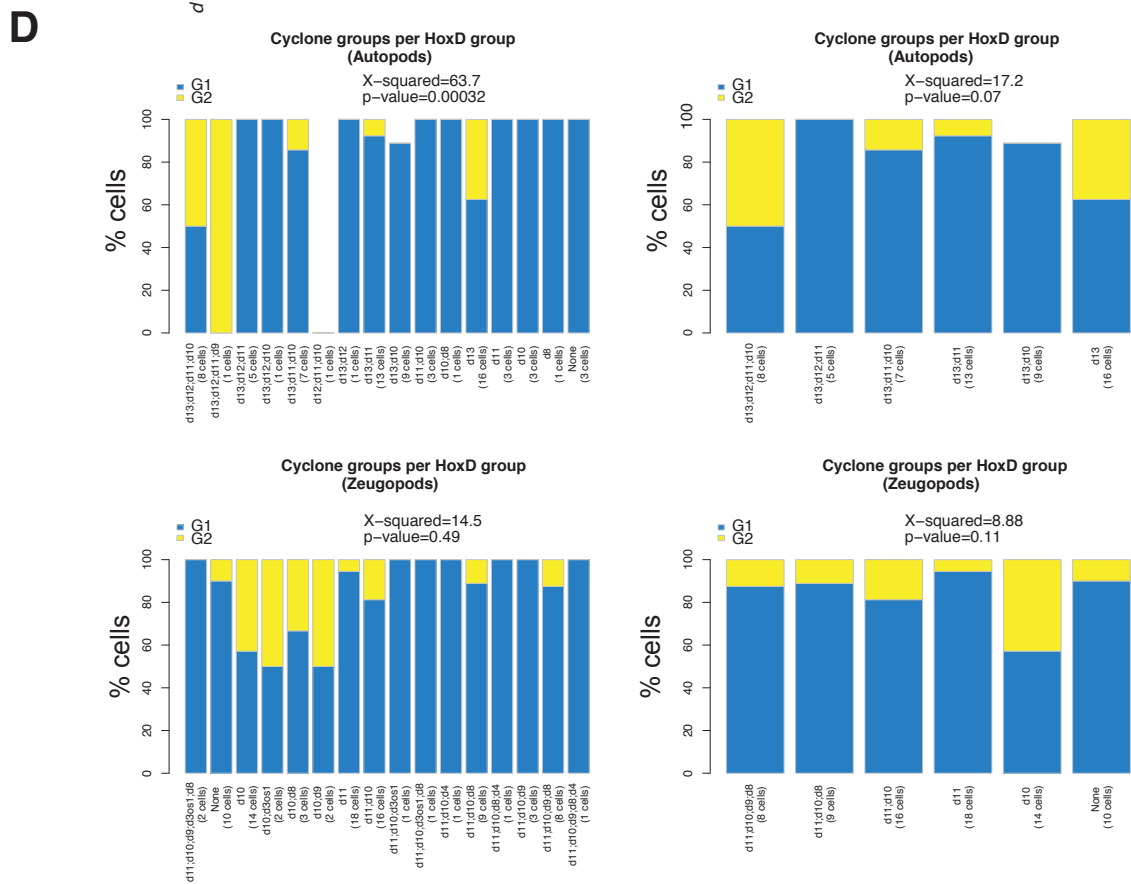

Supplement: Supplementary file 6 — Figure S5. Cyclone analysis of the cell cycle in single cells from autopod and zeugopod. A-B. Graphic representation showing the autopod (A) and zeugopod (B) cells based on their combinatorial expression of Hoxd genes associated with their predicted cell cycle phase as color coded with the above circles in blue (G1), yellow (G2) and green (S phase). C shows the G1 cyclone scores for each of the six main combinations in autopod cells (Right) and zeugopod cells (Left). Error bars represents standard deviation. D. Barplots showing the proportions of G1 and G2 putative state for the cells in all possible combination of posterior Hoxd genes (Hoxd 9 to Hoxd13) observed. (PDF 201 kb) [file 12915_2018_570_MOESM6_ESM.pdf]

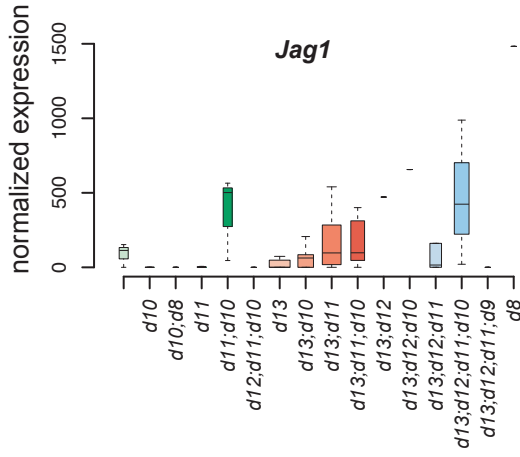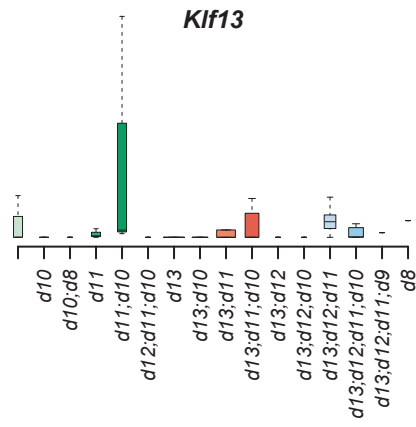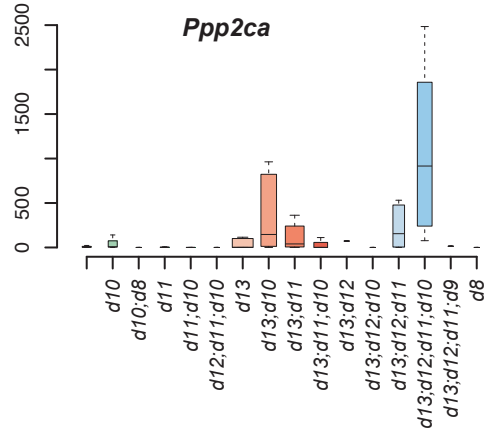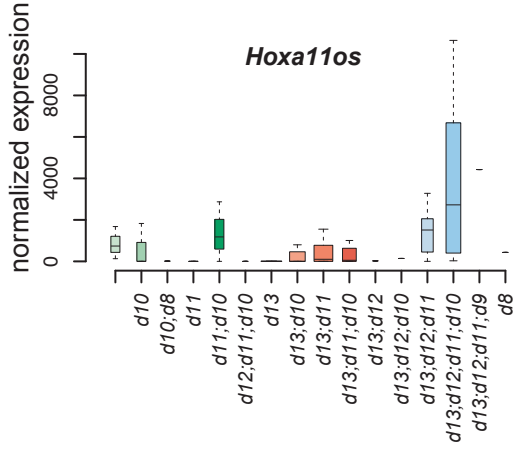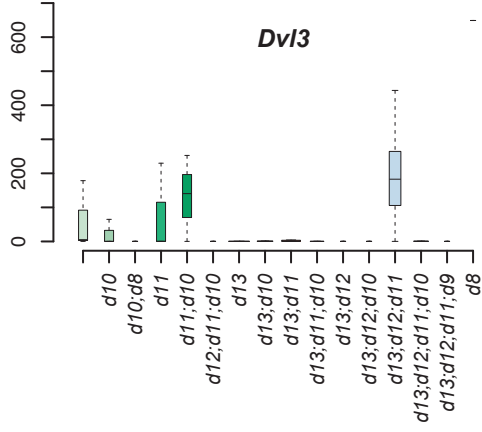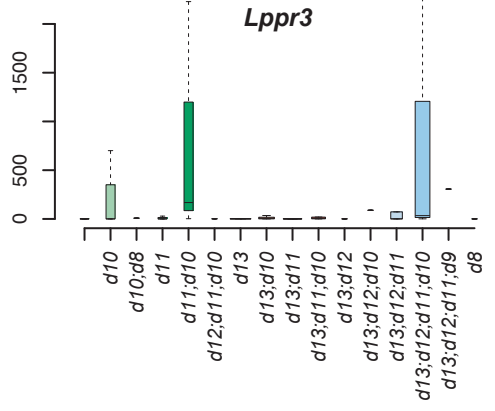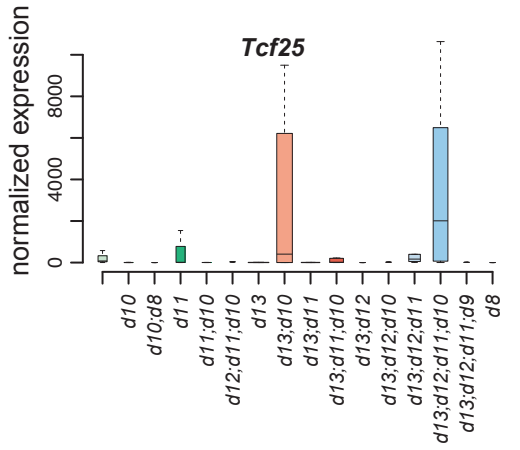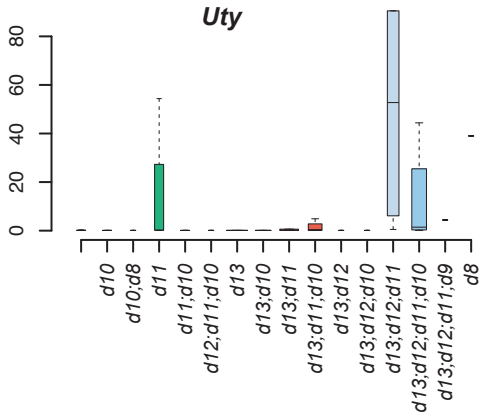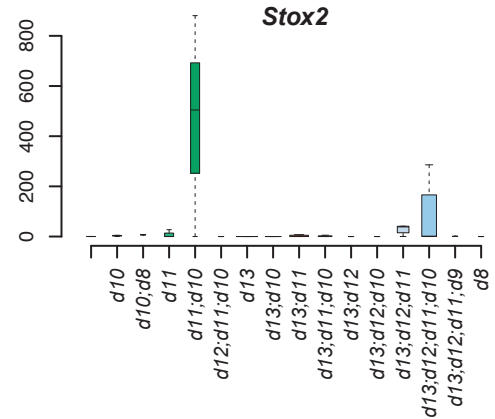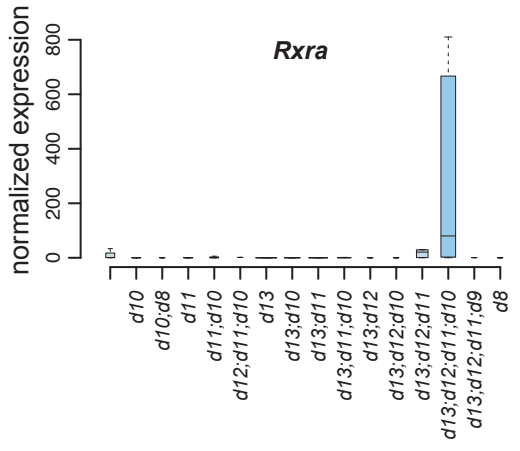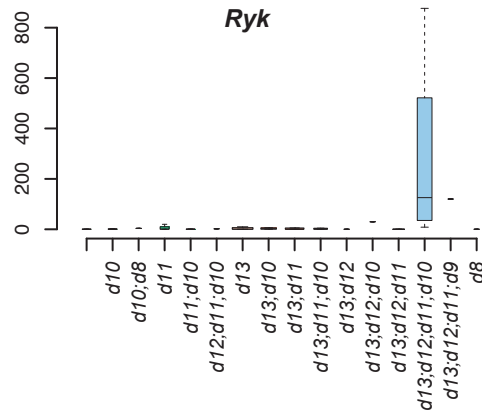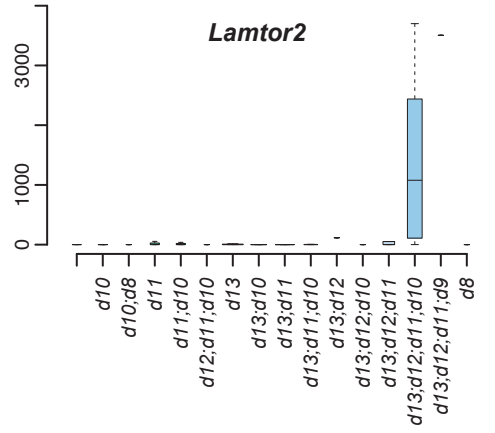

Supplement: Supplementary file 8 — Figure S6. Barplots of expression profiles for twelve representative genes that are differentially expressed between the observed combinations of posterior Hoxd genes in autopod cells. Top rows represent genes expressed in many combinations. Third row shows genes expressed in two or three combinations only. Bottom row shows genes only enriched in the cells expressing Hoxd10 to Hoxd13. (PDF 548 kb) [file 12915_2018_570_MOESM8_ESM.pdf]

**A**

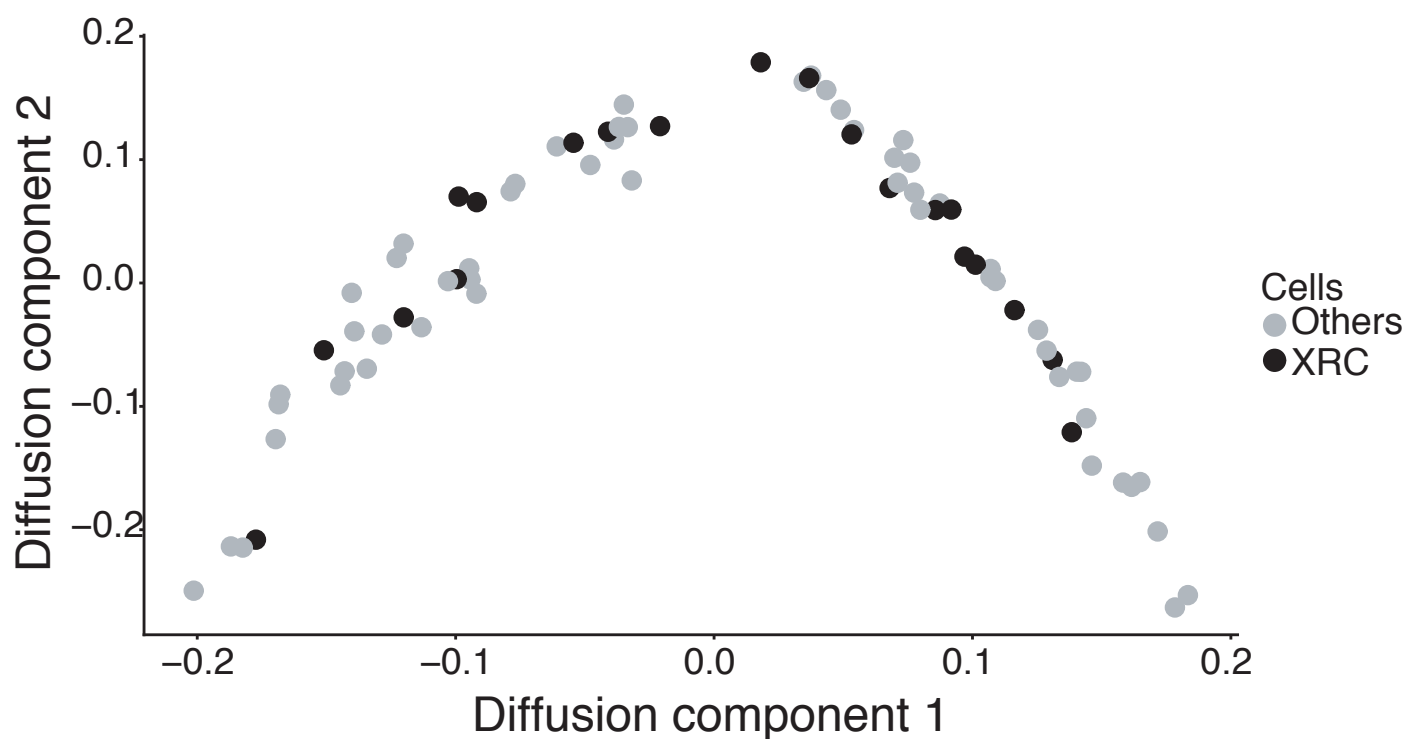

**B**

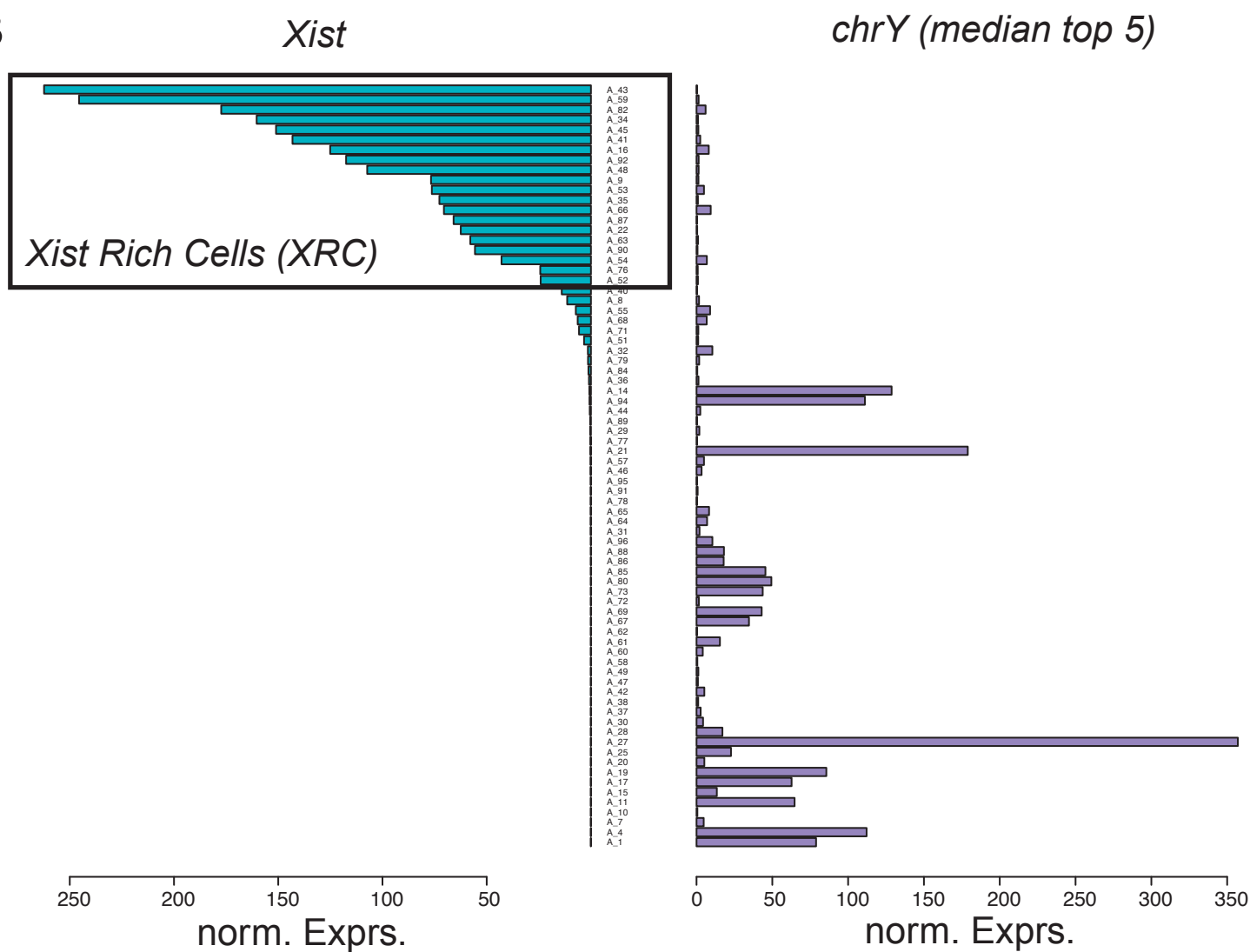

Supplement: Supplementary file 9 — Figure S7. Single embryo analysis (related to Fig. 5). A. The distribution of autopod cells from one female embryo were plotted (black circles) along the pseudotime alignment. The others cells are shown in gray. B. Xist expression levels (green, left) and median expression of the top genes from the Y chromosome (purple, right) were ranked and used to filter the cells originating from one of the four embryos. Cells from this embryo (boxed at the top) are referred to as ‘Xist Rich Cells’ (XRC). (PDF 427 kb) [file 12915_2018_570_MOESM9_ESM.pdf]

**A**

## Autopod cells

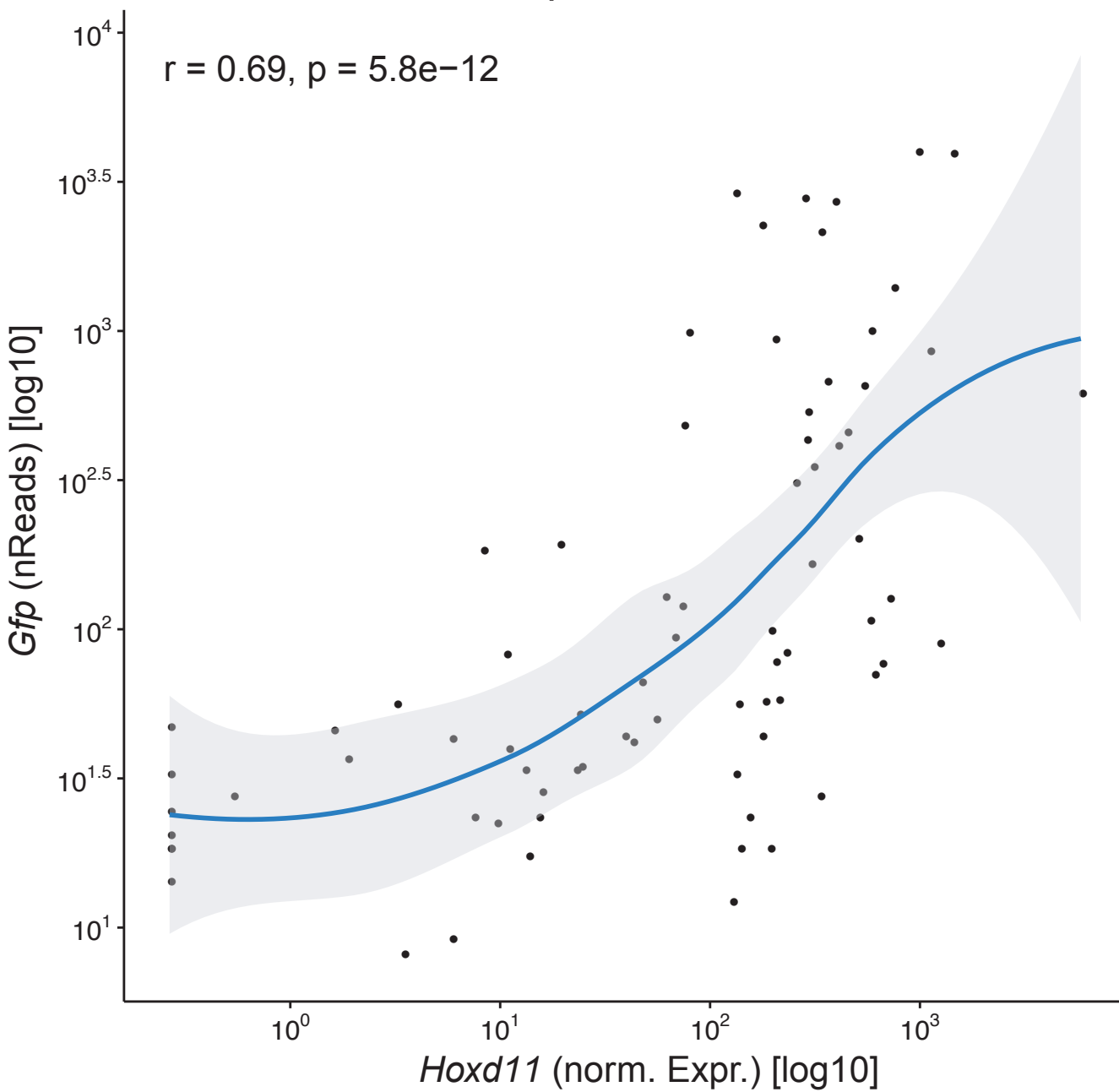**B**

## Zeugopod cells

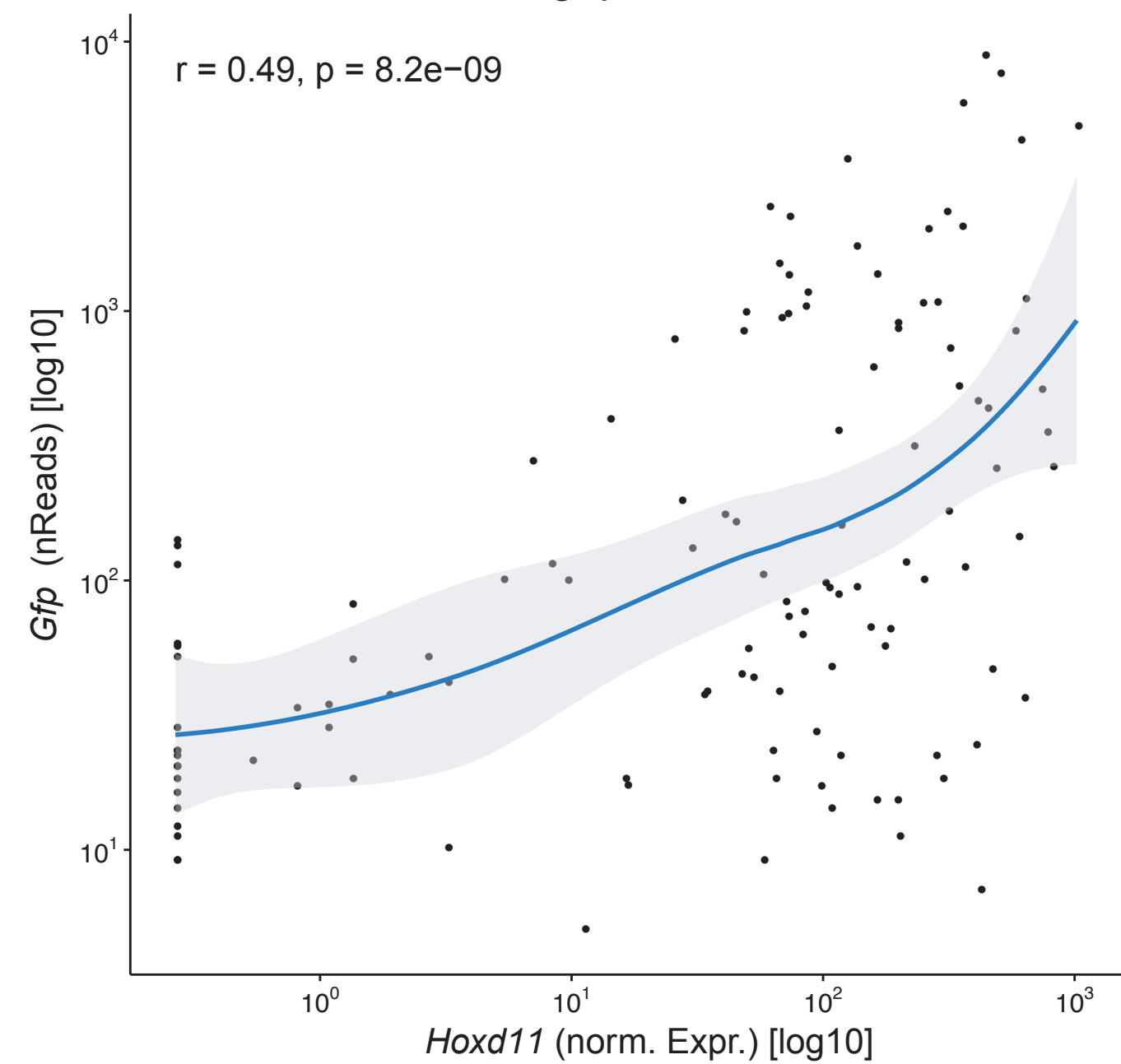

Supplement: Supplementary file 11 — Figure S8. Correlation of expression between the Hoxd11 and Gfp mRNAs. The plots show for every cell the level of Gfp expression (X axis) and Hoxd11 expression (Y axis), dissected either from autopod (A) or from zeugopod (B) tissue. Gene counts from all cells were used to fit a Loess regression curve (blue line) between average scaled gene counts. Pearson correlation tests are shown in the top left of each panel, with r = 0.69 (p = 5.8e−12; A) and r = 0.49 (p = 8.2e−09; B). (PDF 507 kb) [file 12915_2018_570_MOESM11_ESM.pdf]
