# Supplementary material for: Heterogeneous combinatorial expression of Hoxd genes in single cells during limb development
Source: BMC Biol. 2018 Sep 18;16:101. doi: 10.1186/s12915-018-0570-z (PMC6142630; doi:10.1186/s12915-018-0570-z)
Supplement: Supplementary file 2 — Figure S2. Concise description of the methodology and filtering methods used for the single-cell RNA-seq. A. At the top is shown a schematic depicting the Fluidigm workflow from cell dissociation to capture. Below is represented the cassette allowing the expression of GFP under the control of the Hoxd11 endogenous promoter. The bottom left shows the GFP pattern in the E12.5 mouse embryos from which the developing limbs are dissected, sent to FACS and the cells expressing the highest level of GFP proteins captured in the C1 apparatus before libraries are built using a SMARTer kit (steps listed from left to right from left to right). B. Barplots showing the number of mapped reads per cells including the one that map on ERCC endogenous spike-ins (blue) with the number on top of each bar indicating the percentage of these ERCC amongst all reads. C. Cumulative distribution of the number of genes detected amongst all cells with the dotted lines representing the cut-off used to select only the highest qualitative cells. D. Boxplots representing the variation of the number of reads mapped per single cells with an average over 8 million reads per cells in each condition. (PDF 1562 kb) [file 12915_2018_570_MOESM2_ESM.pdf]

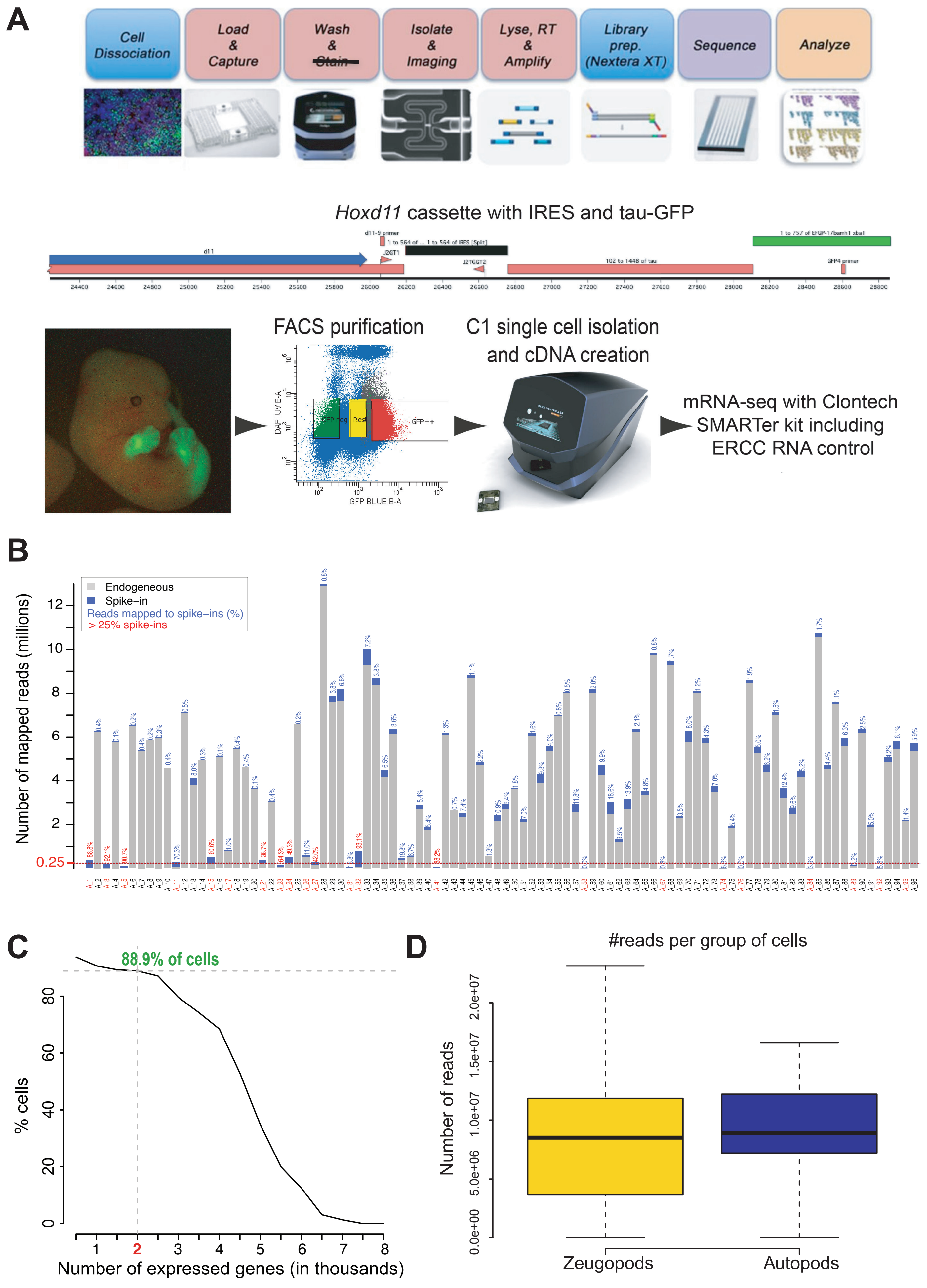
A
Cell Dissociation
Load & Capture
Wash & Stain
Isolate & Imaging
Lyse, RT & Amplify
Library prep. (Nextera XT)
Sequence
Analyze
Hoxd11 cassette with IRES and tau-GFP
FACS purification
GFP neg
Rest
GFP++
DAPI UV B-A
GFP BLUE B-A
C1 single cell isolation and cDNA creation
mRNA-seq with Clontech SMARTer kit including ERCC RNA control
B
Endogeneous
Spike−in
Reads mapped to spike−ins (%)
> 25% spike-ins
Number of mapped reads (millions)
0.25
C
88.9% of cells
% cells
Number of expressed genes (in thousands)
D
#reads per group of cells
Number of reads
Zeugopods
Autopods
