# Supplementary material for: Heterogeneous combinatorial expression of Hoxd genes in single cells during limb development
Source: BMC Biol. 2018 Sep 18;16:101. doi: 10.1186/s12915-018-0570-z (PMC6142630; doi:10.1186/s12915-018-0570-z)
Supplement: Supplementary file 4 — Figure S3. Table of differentially expressed genes between autopod and zeugopod cells. List of the 50 genes with the highest enrichment in autopod cells compared to zeugopod cells from E12.5 Hoxd11::GFP+ developing limb single cells. (PDF 26 kb) [file 12915_2018_570_MOESM4_ESM.pdf]

Top 50 differentially expressed genes between autopod and zeugopod cells

Gene name	Avg Expr	log2 FC	P.Value	adj.P.Val
Hoxd13	3.56	-7.88	1.62E-54	1.74E-50
Msx1	2.39	-4.95	1.76E-24	6.35E-21
Id2	8.57	-4.42	4.03E-13	2.90E-10
Id1	9.69	-3.83	1.41E-09	4.48E-07
Prdm16	3.04	-3.29	8.85E-16	1.36E-12
Lhx9	2.55	-3.16	2.35E-10	9.52E-08
Sox9	6.99	-3.14	1.84E-07	2.65E-05
Msx2	1.61	-2.97	9.99E-15	1.08E-11
Hoxa13	1.72	-2.96	1.27E-19	3.42E-16
Wnt5a	5.25	-2.89	2.36E-08	4.39E-06
Hes1	3.97	-2.73	3.76E-11	2.14E-08
Hoxd12	1.55	-2.72	1.73E-14	1.70E-11
Lgr4	6.15	-2.69	2.15E-06	2.14E-04
Fos	8.4	-2.64	5.31E-05	2.84E-03
Creb5	2.7	-2.59	3.26E-08	5.59E-06
Dusp1	4.45	-2.54	1.17E-05	8.18E-04
Prickle1	2.29	-2.46	3.13E-07	4.08E-05
Twist1	2.61	-2.44	1.28E-08	2.66E-06
Lnp	3.05	-2.44	1.09E-08	2.35E-06
Hnrnpab	5.51	-2.43	4.26E-06	3.49E-04
Id3	11.11	-2.4	1.10E-05	7.76E-04
Cdh11	5.38	-2.4	1.88E-05	1.22E-03
Magoh	6.33	-2.38	1.29E-04	5.37E-03
Jag1	3.2	-2.37	3.04E-06	2.76E-04
Caprin1	3.94	-2.35	2.77E-06	2.56E-04
Mecom	4.15	-2.32	9.15E-06	6.59E-04
Col9a1	3.07	-2.29	2.04E-05	1.31E-03
Prrx2	1.99	-2.24	7.67E-08	1.25E-05
Fgfr2	2.3	-2.19	2.62E-06	2.46E-04
Lhx2	1.33	-2.19	1.86E-11	1.12E-08
Dach1	1.46	-2.17	1.48E-09	4.48E-07
Cdh3	2.08	-2.14	1.04E-08	2.30E-06
Sulf1	2.67	-2.11	2.33E-05	1.46E-03
Trim28	4.9	-2.11	4.44E-04	1.31E-02
Kcnmb4	2.12	-2.09	1.88E-08	3.70E-06
Gli3	4.94	-2.08	5.27E-05	2.83E-03
Timm8b	5.05	-2.07	8.04E-06	5.90E-04
Lmo4	3.12	-2.04	3.62E-06	3.10E-04
Rarg	3.12	-2.02	3.30E-06	2.95E-04
Cdc42ep3	2.55	-1.99	4.73E-05	2.62E-03
Eif3f	3.86	-1.91	1.31E-04	5.41E-03
Aldh1a2	1.46	-1.9	3.17E-06	2.85E-04
Dlx5	1.76	-1.87	2.25E-07	3.11E-05
Nsmce4a	2.1	-1.86	1.58E-05	1.04E-03
Tpbg	2.59	-1.84	3.24E-05	1.91E-03
Egr1	7.17	-1.82	4.20E-03	6.13E-02
Smarcd1	1.99	-1.8	2.19E-06	2.14E-04
